# Novel 2-(Adamantan-1-ylamino)Thiazol-4(5*H*)-One Derivatives and Their Inhibitory Activity towards 11β-HSD1—Synthesis, Molecular Docking and In Vitro Studies

**DOI:** 10.3390/ijms22168609

**Published:** 2021-08-10

**Authors:** Renata Studzińska, Daria Kupczyk, Wojciech Płaziński, Szymon Baumgart, Rafał Bilski, Renata Paprocka, Renata Kołodziejska

**Affiliations:** 1 Department of Organic Chemistry, Faculty of Pharmacy, Collegium Medicum in Bydgoszcz, Nicolaus Copernicus University in Toruń, 2 Jurasza Str., 85-089 Bydgoszcz, Poland; sz.baumgart@cm.umk.pl (S.B.); renata.bursa@cm.umk.pl (R.P.); 2 Department of Medical Biology and Biochemistry, Faculty of Medicine, Collegium Medicum in Bydgoszcz, Nicolaus Copernicus University in Toruń, 24 Karłowicza Str., 85-092 Bydgoszcz, Poland; dariak@cm.umk.pl (D.K.); rafal.bilski@cm.umk.pl (R.B.); renatak@cm.umk.pl (R.K.); 3 J. Haber Institute of Catalysis and Surface Chemistry, Polish Academy of Sciences, 8 Niezapominajek Str., 30-239 Cracow, Poland; wojtek_plazinski@tlen.pl

**Keywords:** 11β-hydroxysteroid dehydrogenase 1, glucocorticoids, metabolic disorders, thiazolone derivatives, molecular docking

## Abstract

A common mechanism in which glucocorticoids participate is suggested in the pathogenesis of such metabolic diseases as obesity, metabolic syndrome, or Cushing’s syndrome. The enzyme involved in the control of the availability of cortisol, the active form of the glucocorticoid for the glucocorticoid receptor, is 11β-HSD1. Inhibition of 11β-HSD1 activity may bring beneficial results for the alleviation of the course of metabolic diseases such as metabolic syndrome, Cushing’s syndrome or type 2 diabetes. In this work, we obtained 10 novel 2-(adamantan-1-ylamino)thiazol-4(5*H*)-one derivatives containing different substituents at C-5 of thiazole ring and tested their activity towards inhibition of two 11β-HSD isoforms. For most of them, over 50% inhibition of 11β-HSD1 and less than 45% inhibition of 11β-HSD2 activity at the concentration of 10 µM was observed. The binding energies found during docking simulations for 11β-HSD1 correctly reproduced the experimental IC_50_ values for analyzed compounds. The most active compound 2-(adamantan-1-ylamino)-1-thia-3-azaspiro[4.5]dec-2-en-4-one (**3i**) inhibits the activity of isoform 1 by 82.82%. This value is comparable to the known inhibitor-carbenoxolone. The IC_50_ value is twice the value determined by us for carbenoxolone, however inhibition of the enzyme isoform 2 to a lesser extent makes it an excellent material for further tests.

## 1. Introduction

According to the World Health Organization (WHO), obesity is one of the most common metabolic diseases. It is also recognized as a global epidemic of the 21st century [1]. The scale of obesity is of interest to many researchers and public health organizations. Obesity is often accompanied by symptoms such as dyslipidemia, arterial hypertension, insulin resistance, or disturbances in carbohydrate metabolism [2]. All these elements make up the definition of the metabolic syndrome [3]. The phenotypic similarity between metabolic syndrome and Cushing’s syndrome, which is characterized by an excess of plasma cortisol, suggests a common mechanism in which glucocorticoids are involved [4,5]. While the molecular basis for Cushing’s syndrome is fairly well understood, the molecular pathomechanism of the metabolic syndrome is still not fully clear, that may pose difficulties in developing therapeutic strategies for this disease. Glucocorticoids have a significant impact on the breakdown of fat stored in the body. Visceral adipose tissue is characterized by a particularly high expression of glucocorticoid receptors, and thus the circulating glucocorticoids have a significant impact on insulin resistance, lipolysis or the expression of adipokines and their release into the liver [6]. These hormones accelerate the differentiation of pre-adipocytes and the accumulation of adipocytes in adipose tissue [7]. Despite many similarities to Cushing’s syndrome, normal and sometimes even lower levels of glucocorticoids are observed in the metabolic syndrome [8]. This is due to the action of the intracellular enzyme 11β-hydroxysteroid dehydrogenase (11β-HSD1).

Cortisol, which is one of the main glucocorticoids (GCs) secreted by the cortex of the adrenal gland, helps maintain homeostasis. Its concentration increases during times of stress. In addition, it is involved in the regulation of carbohydrate, lipid, and protein metabolism. It causes the release of fatty acids and also increases the number of neutrophils or platelets. It also shows anti-inflammatory properties [9].

In 1953, it was proved that the conversion of cortisol in humans is related to the activity of the 11β-HSD enzyme. Its presence was also confirmed in various tissues, including in the kidneys or placenta. Subsequent studies showed that the liver converts cortisone into cortisol, which led to the hypothesis of the existence of two isoforms of this enzyme, which were described in the late 1990s [10,11]. 11β-HSD1 is a product of the HSD11B1 gene, which is located on chromosome 1. It is expressed in many human tissues with high sensitivity to glucocorticoids, including adipose tissue, liver, brain, gonads, and vessels. Its role is to increase the concentration of the active form of glucocorticoids in the tissue, which leads to the activation of the glucocorticoid receptor. In obesity-related disorders, the role of 11β-HSD1 is related to the increase in peripheral cortisol clearance, which results in the normal concentration of cortisol in the blood with its increased production in obese people [12].

11β-HSD1 activity is elevated in subcutaneous adipose tissue in obese patients [13,14,15]. In turn, Prasad et al. observed the decreased activity of this enzyme in the liver of obese rats in their research [16]. Scientific reports also confirm that inhibition of 11β-HSD1 activity brings beneficial results for the alleviation of the course of the metabolic syndrome. Research by Schnackenberg et al. and Shao et al. proved that in obese rats, administration of the 11β-HSD1 inhibitor for four weeks led to a reduction in blood pressure, insulin resistance and a decrease in blood triglyceride levels by affecting the secretion of IL-6, TNF-α, and adiponectin [17,18].

The above reports indicate that the search for selective 11β-HSD1 inhibitors may support the therapeutic process in patients with obesity and the metabolic syndrome.

Heterocycles including nitrogen and sulfur have been investigated for a long time because of their synthetic diversity and therapeutic importance. Among a wide variety of heterocyclic compounds, thiazoles and their derivatives are favored candidates for the synthesis of pharmaceuticals [19].

**Figure 1:**
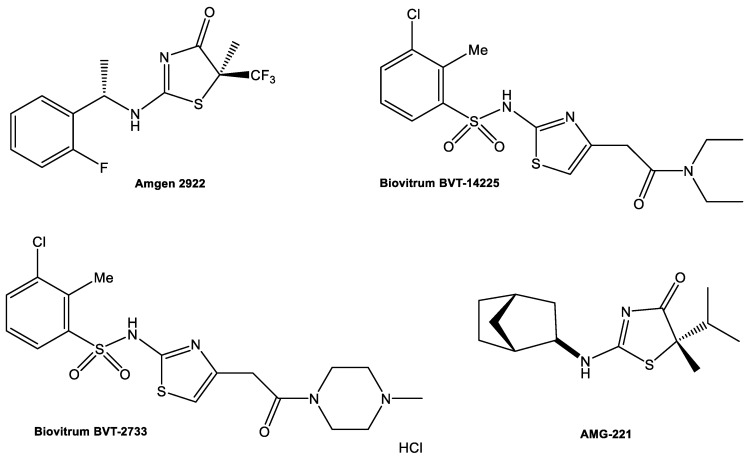
11β-hydroxysteroid dehydrogenase type 1 inhibitors.

Compounds containing dihydrothiazoles in their structure exhibit a broad spectrum of biological activity, including antiproliferative, antibacterial, anti-inflammatory, antiparasitic, and antifungal activity [20,21]. Thiazole derivatives are also found as new enzyme inhibitors, for example Biovitrum BVT-2733, Biovitrum BVT-14225, AMG-221, and Amgen 2922 can inhibit 11β-hydroxysteroid dehydrogenase type 1 (Figure 1) [22,23,24,25,26,27,28,29].

**Figure 2:**
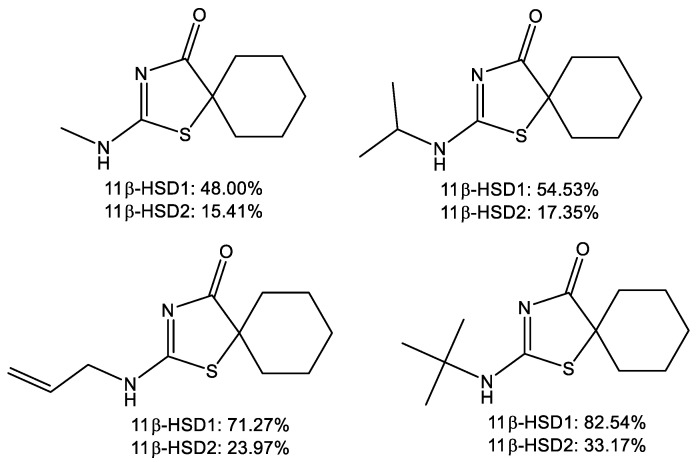
Thiazolone derivatives as selective inhibitors of the 11β-HSD1.

Our previous research has also shown that some of the thiazolone derivatives may be selective inhibitors over one of the 11β-HSD isoforms (Figure 2) [30,31,32,33,34].

On the other hand, a group of compounds of interest from the pharmacological point of view includes the adamantane (tricyclo [3.3.1.13.7]-decane) derivatives. The introduction of the bulky and lipophilic adamantyl groups reduces the lability of the molecule and allows a better fit in the hydrophobic receptor/catalyst pocket, which may be directly related to biological activity. The adamantyl group positively modulates the therapeutic index of many experimental compounds increasing drug-like qualities of a lead compound, without increasing toxicity [35].

Several classes of selective, non-steroidal adamantyl-based 11β-HSD1 inhibitors have been published. These include adamantine triazoles such as the Merck compound 544 [36], amides from Abbott [37,38], sulfone, sulfonamide [39], pyrrolidine carboxamide [40], and adamantyl ethanone derivatives [41].

For this reason, we focused our attention on derivatives containing both the thiazolone ring and the adamantyl group by synthesizing a series of new 2-(adamantylamino)thiazol-4(5*H*)-one derivatives as potential 11β-HSD1 inhibitors.

## 2. Results and Discussion

### 2.1. Chemistry

2-(Adamantan-1-ylamino)thiazol-4(5*H*)-one derivatives were obtained by reacting 1-(adamantan-1-yl)thiourea with 2-bromo esters (Table 1). The reactions were carried out under various conditions depending on the type of the substituent in bromo ester. We initially attempted to prepare all the described compounds in chloroform at room temperature. The synthesis of compounds **3a**–**3d** with simple alkyl substituents (or no substituent) at C-5 was promising, the yield of the reaction was 60.1–75.8%. In the accordance with the same procedure, the reaction of 1-(adamantan-1-yl)thiourea with esters containing aromatic substituents allowed obtaining compounds **3g** and **3h**, with good (66.2%) and moderate (25.5%) yields, respectively. Unfortunately, under these conditions, the synthesis of **3e**–**3f** derivatives with branched substituents at C-5 did not give the expected results. TLC analysis showed the formation of trace amounts of products, even within long reaction times (the progress of the reaction was monitored for 4 months). Therefore, these compounds were obtained by heating under reflux in an alkaline medium (sodium methoxide). The change of the reaction conditions allowed obtaining compounds **3e**–**3f** with isolated yields of 15.8–18.7%. Our previous research on the synthesis of 2-aminothiazol-4(5*H*)-one derivatives containing the *spiro* thiazole and alicyclic ring system showed that compounds of that type could be obtained by prolonged heating of the reactants in ethanol in the presence of *N*,*N*-diisopropylethylamine [30,31,32,33]. After 7 days for **3j** and 14 days for **3i**, a complete conversion of substrate to product was observed. The low isolated efficiency is the result of the losses due to the difficulty in purifying the product from diisopropylethylamine.

### 2.2. In Vitro Studies

The obtained 2-(adamantan-1-ylamino)thiazol-4(5*H*)-one derivatives were tested in vitro for the inhibition of two isoforms of 11β-hydroxysteroid dehydrogenase: 11β-HSD1 and 11β-HSD2. All the synthesized compounds at a concentration of 10 µM inhibited the activity of isoform 1 in the range of 15.30 to 82.82% (Table 2). For most of them, over 50% inhibition of 11β-HSD1 activity at the concentration of 10 µM was observed (except for compounds **3g** and **3h** with aromatic substituents in the 5-position of the thiazole ring and unsubstituted compound **3a**). The most active compound **3i** (IC_50_ = 0.31 µM) contained the cyclohexane substituent at the 5-position of the thiazole ring in the *spiro* system. The inhibition of activity of isoform 1 by this compound is comparable to the known inhibitor carbenoxolone. When cyclohexane substitute was replaced with cyclobutane ring, a slight decrease in inhibition was observed, down to 74.13% (IC_50_ = 3.32 µM). High percent inhibition (76.40–69.22%) was also shown by derivatives containing isopropyl (**3e**), propyl (**3d**), and ethyl (**3c**) substituents, for which IC_50_ was 1.19, 3.23, and 5.44 µM, respectively. On the basis of the obtained results, it can be concluded that in comparison with the derivatives containing chain substituents at the nitrogen atom [30,31,32,33], 2-(adamantan-1-ylamino)thiazol-4 (5*H*)-ones show a higher activity towards the inhibition of 11β-HSD1. In vitro studies showed that all the derivatives obtained also inhibit the activity of enzyme isoform 2, yet to a lesser extent (all analyzed compounds inhibited the activity of 11β-HSD2 by less than 50%). Compounds **3b** and **3i** happened to be the most active, with 44.71% inhibition at the concentration of 10 µM. Only for derivatives **3a** and **3h**, the percent inhibition of isoform 2 was higher than that of isoform 1. There was a slight difference in the inhibition of the activity of both isoforms for compound **3a** (29.81% for 11β-HSD2 vs. 22.27% for 11β-HSD1) whereas for compound **3h** the difference in inhibition of these isoforms was quite significant (41.83% for 11β-HSD2 vs.15.30% for 11β-HSD1). It is the substance with the highest inhibition selectivity in relation to isoform 2 in this series of compounds. Analyzing the obtained results of in vitro studies, it can be concluded that compound **3i** is the most interesting from the point of view of regulating the level of cortisol, characterized by a high degree of inhibition of 11β-HSD1 (comparable to the known inhibitor-carbenoxolone) and a large difference in inhibiting the activity of isoforms 1 and 2. Note that although compound **3i** at a concentration of 10 µM inhibits the activity of isoform 2 by more than 44%, it is still a lower value than that obtained for carbenoxolone. These results allow outlining future prospects of using this inhibitor as a potential drug in the treatment of such diseases as Cushing’s syndrome, metabolic syndrome or type 2 diabetes. Therefore, it is worth subjecting it to further tests.

### 2.3. Molecular Docking

**Figure 3:**
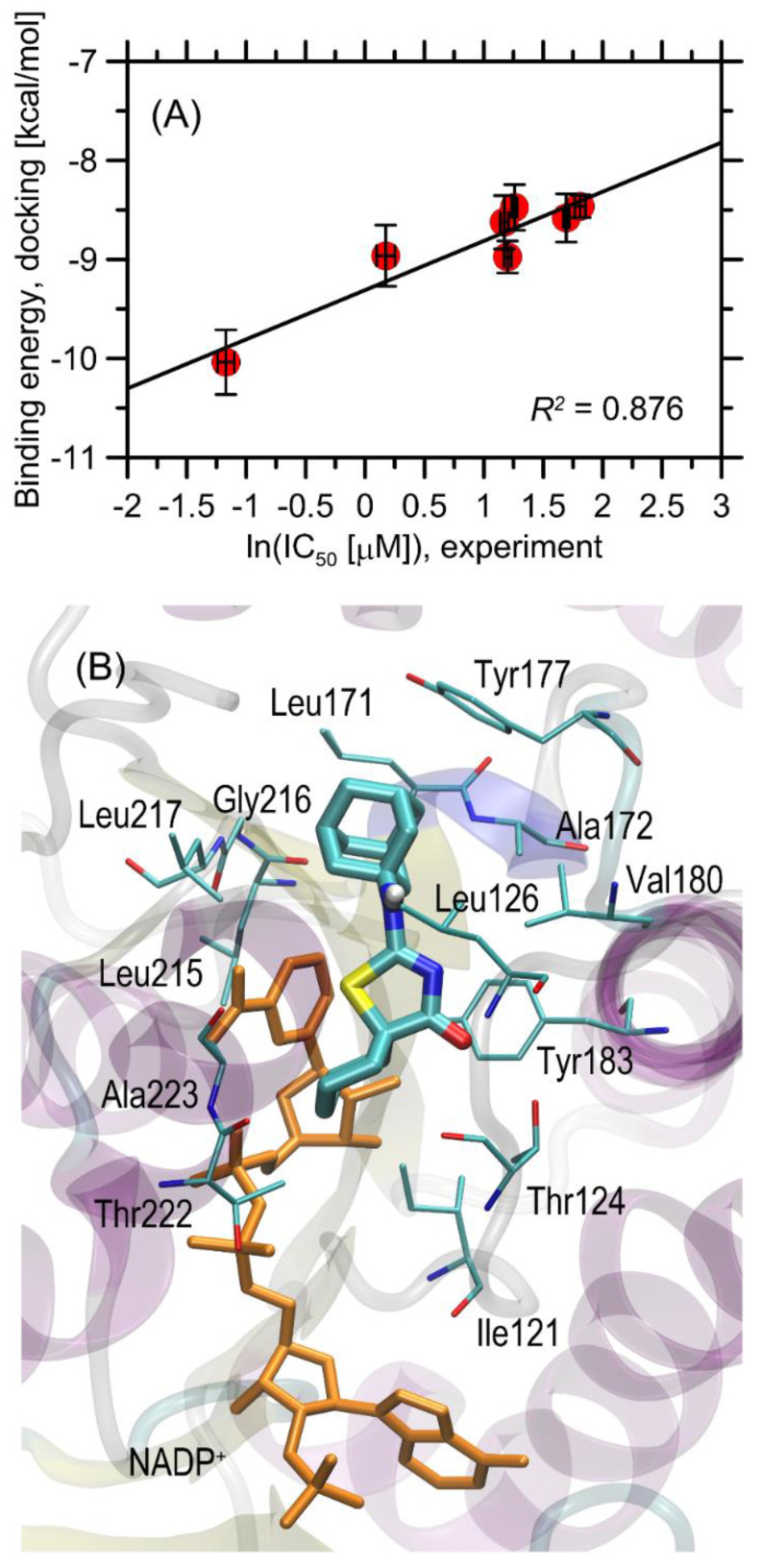
(**A**) The correlation between the binding energies calculated for 11β-HSD1 protein interacting with a set of ligand molecules with the experimentally inferred IC_50_ values. The numerical values can be found in Table 2. (**B**) The energetically favorable location of the **3i** ligand molecule bound to the 11β-HSD1 (pdb:3czr) structure. The ligand molecule is shown as thick sticks, whereas all the closest amino-acid residues (<0.38 nm) are represented by thin sticks. Orange sticks represent the NADP^+^ molecule, also present in the protein crystal structure. The description of the interaction types is given in the text.

The binding energies found during docking simulations are given in Table 2 and are graphically illustrated in Figure 3A. The magnitude of the determined binding energies obtained for considered ligands, varies within a relatively narrow range of ~−7.9–−10.3 kcal/mol. Roughly the same magnitude of binding energies (−9.0–11.1 kcal/mol) was found during docking of ligands originally bound to the protein structures used in the study. When considering the stereoselectivity effects that may influence the binding strength, it was observed that the binding energy corresponding to a given pair of stereoisomers, differs very slightly, by not more than 0.3 kcal/mol. Thus, it can be concluded that the stereoconfiguration of the chiral ligand does not affect its binding affinity. The order of binding energies correctly reproduces the experimental IC_50_ values for those compounds for which this parameter was measured (i.e., all except for **3a**, **3g**, and **3h**). This includes the prediction of the highest binding energy for the most potent compound (**3i**). Regarding the complete set of compounds, the correlation of ln (IC_50_) vs. binding energy is apparent and the associated correlation coefficient is equal to 0.876. However, the binding energies of 2 out of 3 compounds for which IC_50_ > 10 µM (**3g**, **3h**) do not follow this trend. The remaining compound, **3a**, exhibits the weakest binding strength among all studied ligands (−7.91 kcal/mol), which explains its limited inhibition properties. On the other hand, it is not the case of **3g** and **3h** which display very high binding energies (~−10 kcal/mol) but their IC_50_ >10 µM. This apparent disagreement between theoretical predictions and experiment can be explained by more detailed analysis of the ligand–protein interactions and the arrangement of the ligand molecules in the binding cavity (see the discussion in the subsequent paragraph).

In view of a satisfactory agreement between the theoretical and experimental results, we decided to perform some more detailed analysis, focused on identifying the structural aspects of ligand–protein interactions. In parallel to binding energies, the results of the docking studies can also be analyzed with respect to the mechanistic interaction patterns that may be significant in the context of interpretation of the obtained binding energy values and the measured properties. The summary given below relies on analyzing the ligand–protein contacts that occur if the distance between any corresponding atom pair is smaller than the arbitrarily accepted value of 0.38 nm. The latter value has been chosen on the basis of the van der Waals radius of the largest atom present in the studied systems (i.e., Br). Although Br atoms are not present in all systems, we kept this limiting value for the sake of consistency.

**Figure 4:**
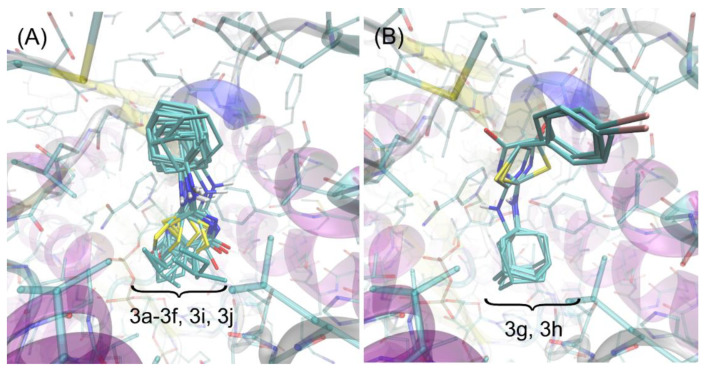
The superposition of poses of all ligands found during docking study. (**A**) The cluster of poses characteristic for the most energetically favorable orientations of ligands **3a**–**3f**, **3i**, and **3j**. (**B**) The alternative poses exhibited by compounds **3g** and **3h** (see discussion in the text).

We have found that the majority of the studied ligands dock to the protein structure in a very similar manner (see Figure 4A). Their orientation in the binding cavity closely resembles that characteristic of another group of structurally related ligands, considered in our previous study [30]. The alternative poses are associated with notably higher energy levels (by at least 0.8 kcal/mol). The similarity of the docking poses also includes the stereoisomers of the same compound. The two compounds (**3g** and **3h**) exhibit the alternative binding mode, significantly differing in both the molecular conformation of the ligand itself and the ligand–protein set of contacts (Figure 4B). The alternative poses characteristic of **3g** and **3h**, structurally closer to those displayed by remaining ligands, also exist but are associated with less favorable binding energies (higher by > 0.7 kcal/mol). Let us note that **3g** and **3h** are the same compounds for which IC_50_ > 10 µM were found and, at the same time, high binding energies during docking studies were obtained. This apparent inconsistency may be explained by taking into account the diverse binding patterns shown by these compounds. It seems that only a certain type of ligand arrangement in the binding cavity is correlated with its inhibition properties and such arrangement is characteristic of compounds **3a**–**3f**, **3i**, and **3j**, as well as by large cohort of other compounds studied previously [30]. In spite of high binding energies, such properties are not exhibited by either **3g** or **3h**. Elucidating detailed mechanisms lying behind this observation is outside the scope of this paper.

The detailed description of the protein–ligand contact pattern is provided below. It relies on the most potent compound **3i**. However, as mentioned above, the found interactions pattern is representative of all studied compounds that display some inhibition properties. The graphical illustration of the docking results is given in Figure 3B.

The bulky, aliphatic moiety present in all studied ligands maintains close contacts with a series of aliphatic sidechains of Ala172, Leu217, Leu215, Leu171, Leu126, and Val180. Such placement of the aliphatic group in the center of aliphatic ‘core’ created by above-mentioned amino acid residues is energetically favorable due to hydrophobic interactions and minimizing the hydrophobic surface of cavity, exposed to the contact with water. Other contacts involving the same bulky, aliphatic moiety are created by Gly216 and Tyr177. In the latter case, the presence of H–π stacking and the associated attractive interactions can be concluded. On the other hand, the proximity of Gly216 is probably the opportunistic consequence of orientational preferences dictated by other, stronger types of interactions. Interestingly, the amine moiety of the ligand displays no well-defined attractive interaction (such as hydrogen bonding of H–π stacking) with any of the neighboring amino acid residues. This is common for all studied ligands and can be seen in Figure 4 where the conformational scatter of this group can be observed. The central part of the ligand molecule, i.e., the thiazole ring, interacts via π–π interactions with the neighboring aromatic moieties belonging to either NADP^+^ molecule or Tyr183. The non-negligible spatial fluctuations of this moiety across all groups of compounds (see Figure 4) let us speculate that these interactions may have an interchangeable character. However, in some of the cases (e.g., for **3i**), there appears additional, attractive interaction, namely, hydrogen bonding between carbonyl oxygen atom attached to the thiazole ring and the sidechain of Thr124. Finally, the diverse group of substituents attached to the thiazole ring, of type varying from one ligand to another, exhibits contacts with a limited number of amino acid residues, including the ribose ring of NADP^+^, Ala223, Thr222, Thr124, and Ile121. In the case of Ile and Ala, those contacts have a character of hydrophobic interactions, whereas it is hard to unequivocally distinguish any specific interactions involving the remaining two residues. In spite of relatively small number of possible contacts, they are apparently sufficient to discriminate between various binding affinities observed either experimentally or theoretically. Finally, the proximity of this moiety with the rigid backbone fragments of Ala223 and Thr222 also explains why **3g** and **3h** exhibit the alternative binding pattern; the large dimension of their substituents (phenyl groups) prevents to accommodate them in the arrangement analogous to that displayed by remaining ligands.

Summarizing, the driving force for binding seem to be the hydrophobic interactions of the ligand with leucines and Tyr177, supported by the π–π interactions with Tyr183 and NADP^+^, whereas divergences between binding energies across the whole group of compounds can be ascribed to the interactions between the substituent attached to the thiazole ring and the region of the protein located in the vicinity of Thr124 and Thr222.

There exist several structurally similar ligands for which the binding poses have been resolved experimentally by crystallographic studies. The poses of ligands identified during docking procedure are much closer to those identified for structurally related ligands reported in [42] in comparison to those studied in [43]. In particular, the following analogies between ligand–protein contacts can be noticed in the case of compound 4k investigated in [42]: (i) the bulky adamantyl moiety, present in both our compounds and 4k maintains contacts with the same set of amino acid residues: Leu171, Tyr177, Gly216, Leu217, Val180, and Leu215. Leu126 is slightly more distant in comparison to our results. (ii) There is no thiazole moiety in compound 4k, however, its role (i.e., attractive H–π interactions with Tyr183 and NADPH) is played collectively by the two chemically-related ring moieties located in central part of the ligand. (iii) The methoxy group of 4k plays the role of R1 and R2 substituents, present in our compounds and interacts with Ile121, Thr222 and Thr124. Interestingly, even relatively small chemical alteration of such ligand leads to drastic changes in its orientation in the binding cavity, as discussed in detail in [42]. More precisely, the hydroxylation of the adamantyl group triggers the reorientation of the ligand according to which the hydroxyadamantyl group interacts with Tyr183, Thr124, and NADPH and the central fragments of ligand molecule with Leu126. The analogous orientation of the hydroxyadamantyl-containing ligands has also been reported in [43]. Finally, such orientation is close to the arrangements found in our study for compounds **3g** and **3h**. It is not entirely clear why in the latter case such alternative poses are correlated with reduced inhibition properties, but one can notice the lack of certain attractive ligand–protein interactions which are reported in refs. [42,43] but not in our results and, therefore, may be crucial in this context. Namely, unlike the cocrystalized ligands, **3g** and **3h** compounds do not exhibit hydrogen bonding with Thr124, Asp259, or Tyr177.

### 2.4. Bioavailability and Alerts for PAINS—In Silico Simulation

Physicochemical parameters were calculated for the synthesized 2-(adamantan-1-ylamino)thiazol-4(5*H*)-one derivatives (**3a**–**3j**) to evaluate their probable bioavailability after oral administration. The availability after oral administration was evaluated according to the two most important rules used in medicinal chemistry, namely Lipinski’s rule (MW ≤ 500 Da; LogP ≤ 5; nOHNH ≤ 5; nON ≤ 10) and Veber’s rule (tPSA ≤ 140 A^2^; Nrotb ≤ 10) [44,45]. The parameters needed for evaluation were calculated using Molinspiration program and are presented in Table 3 [46].

Analysis of the obtained parameters against the descriptors of the above rules showed that only compound **3h** does not satisfy Lipinski’s rule of five (LogP > 5). The other nine derivatives (**3a**–**3g, 3i**–**3j**) did not show violations against the presented rules, which means that they will probably be characterized by good bioavailability after oral administration.

In medicinal chemistry, in addition to predicting oral bioavailability, an important aspect is also the design of the compound penetrating the selected target tissue. A particularly interesting ability of the compound is to penetrate the blood–brain barrier. It is likely that good penetration into the cerebrospinal fluid should be associated with such physicochemical parameters as tPSA < 90 A^2^, molecular weight < 450 Da, nOHNH < 3, and LogP 2–5 [50]. Among the 2-aminothiazol-4(5*H*)-one derivatives presented in the publication, only the compound **3h** exceeds one assumption for LogP = 2–5 and, unlike the other derivatives of the series, it will probably not have an effect on the central nervous system.

Moreover, we conducted the tests to assess whether the compounds are PAINS (Table 3) [47,48,49]. The results of these screening studies indicate that all tested compounds are not matching PAINS, which means that they do not contains potentially problematic fragments which would give false-positive biological output.

## 3. Materials and Methods

### 3.1. General Information

^1^H- and ^13^C-NMR spectra (Figures S1A–J and S2A–I)—the Bruker Avance 400 and 700 apparatus (TMS as an internal standard).

HRMS (high-resolution mass spectrometry)-Synapt G2 Si mass spectrometer (Waters). The measurement results were processed with MassLynx 4.1 software (Waters) (Figure S3A–J).

### 3.2. Reagents and Solvents

Solvents: chloroform, diethyl ether, dimethylsulfoxide, ethyl acetate, ethyl alcohol, hexane, methyl alcohol (Avantor Performance Materials Poland S.A., Gliwice, Poland).

Reagents for synthesis: *N*-1-Adamantylthiourea 97% (Fluorochem, Hadfield, United Kingdom), 2-bromo esters: ethyl 2-bromopropionate 99%, 2-bromobutyrate 98%, 2-bromovalerate 99%, 2-bromo-3-methylbutyrate 95%, 2-bromoisobutyrate 98%, 2-bromophenyl acetate 97%, 2-bromo(4-bromophenyl) acetate 97%, bromocyclobutane carboxylate 95% and methyl 1-bromocyclohexane carboxylate 97%-(Alfa Aesar, Kandel, Germany, Acros Organic Geel Belgium, Sigma-Aldrich Poznań Poland).

Auxiliary reagents: *N*-ethyldiisopropylamine 99% (Alfa Aesar, Kandel, Germany), hydrochloric acid, magnesium sulfate, sodium, and sodium hydroxide (Avantor Performance Materials Poland S.A., Gliwice, Poland).

TLC and column chromatography: 5 × 10 cm silica gel TLC plates coated with F-254 (Merck, Darmstadt, Germany).

Column chromatography: silica gel MN kieselgel 60M with 0.04–0.063 mm grain diameter (Macherey-Nagel, Oensingen, Switzerland).

11β-HSD1 assays: carbenoxolone (sodium salt) (Cayman Chemical Company, Ann Arbor, MI, USA), cortisone, NADPH tetrasodium salt, phosphate buffer powder, (Sigma-Aldrich, Poznań, Poland), Pooled human liver microsomes, mixed gender, 1 mL, 20 mg/mL Lot No.1410013-XenoTech, Cortisol Elisa Ref DkO001 Lot No. 4715A (DiaMetra, Spello, Italy), ELISA Kit for 11-Beta-Hydroxysteroid Dehydrogenase Type 1 Lot No.L160706125-(Cloud-Clone Corp., Wuhan, China), PBS Lot No. H161008 (Pan Biotech, Aidenbach, Germany).

11β-HSD2 assays: 18-beta-glycyrrhetinic acid-(Acros Organic, Geel, Belgium), cortisone, NAD cofactor, phosphate buffer powder (Sigma-Aldrich, Poznań, Poland), Human Kidney Microsomes, mixed gender, 0.5 mL, 10 mg/mL Lot No. 1710160 XenoTech, Cortisol Elisa Ref DkO001 Lot No. 4715A-(DiaMetra, Spello, Italy), Enzyme-Linked Immunosorbent Assay (ELISA) Kit for 11-Beta-Hydroxysteroid Dehydrogenase Type 2 Lot No. L191113457-(Cloud-Clone Corp., Wuhan, China), PBS Lot No. H161008 (Pan Biotech, Aidenbach, Germany).

### 3.3. General Procedures of Synthesis

All reactions were controlled by TLC chromatography (hexane: ethyl acetate 1:1).

#### 3.3.1. Method A (Synthesis of Compounds **3a**–**3d** and **3g**–**3h**) 

*N*-adamantylthiourea (**1**) (1.25 mmol (0.262 g)) and 1.37 mmol of appropriate 2-bromo ester (**2a**–**2d**, **2g**–**2h**) were dissolved in 15 mL of chloroform and stirred at room temperature for 7 days (except for **2d** and **2g**, which were stirred for 10 and 14 days, respectively). The obtained solids **3a**–**3d** and **3g**–**3h** were collected by filtration and purified by crystallization from ethanol [30,31,32,33].

#### 3.3.2. Method B (Synthesis of Compounds **3e**–**3f**) 

Sodium (0.25 mmol (0.057 g)) was added to 5 mL of anhydrous methanol. Next, 1.25 mmol (0.262 g) of *N*-adamantylthiourea (**1**) and 1.37 mmol of appropriate 2-bromo ester (**2e**–**2f**) were added. The obtained mixture was heated for 7 days (**2e**) or 14 days (**2f**). Then, after evaporation of methanol, the crude solid was dissolved in 10 mL of water and neutralized by 2M hydrochloric acid to pH~7–8. Products **2e**–**2f** were extracted by chloroform (4 × 15 mL), then dried by MgSO_4_ and filtered out. After evaporation of chloroform, obtained mixtures were purified by preparative chromatography plate using (hexane:ethyl acetate 1:1). Compounds **3e**–**3f** were washed out by chloroform [51].

#### 3.3.3. Method C (Synthesis of Compounds **3i**–**3j**) 

*N*,*N*-diisopropylethylamine (5.5 mmol (0.935 mL)), 50 mmol (1.05 g) of *N*-adamantylthiourea (**1**) and 5.5 mmol of bromo ester **2i** or **2j** were added to 5 mL of 99.8% ethanol. The obtained mixture was heated for 7 days (**2j**) or 14 days (**2i**), then the solvent was evaporated. The crude product **3i** was purified by column chromatography, while **3j**, by crystallization from ethanol [30,31,32,33].

2-(adamantan-1-ylamino)thiazol-4(5*H*)-one (**3a**)—Yield: 64.1%. M.p. 271–273 °C. ^1^H-NMR (700 MHz, CDCl_3_, δ ppm, J Hz): 12.11 (br, 1H, NH), 3.97 (s, 1H, C^5^-H), 2.24 (s, 3H, Ad), 2.17 (s, 6H, Ad), 1.73 (dd, 6H, Ad, 12.0 32.8). ^13^C-NMR (100 Hz, CDCl_3_, δ ppm): 40.31_B_ (3C, C_10_H_15_), 34.98_C_ (3C, C_10_H_15_), 28.82_D_ (3C, C_10_H_15_). HR-MS *m*/*z* 251.1227 [M^+^ + 1] (calculated for C_13_H_19_N_2_OS: 251.1218). R_f_ (silicagel, AcOEt:hexane 1:1): 0.23.

2-(adamantan-1-ylamino)-5-methylthiazol-4(5*H*)-one (**3b**)—Yield: 75.8%. M.p. 265–267 °C. ^1^H-NMR (700 MHz, CDCl_3_, δ ppm, J Hz): 11.88 (br, 1H, NH), 4.30 (q, 1H, C^5^-H, 7.0), 2.22 (s, 3H, Ad), 2.16 (s, 6H, Ad), 1.75 (d, 3H, CH_3_, 7.0), 1.72 (dd, 6H, Ad, 12.7 29.3). ^13^C-NMR ^13^C-NMR (100 Hz, CDCl_3_, δ ppm): 170.89 (C-4), 169.87 (C-2), 58.75_A_ (1C, C_10_H_15_), 44.34 (C-5), 40.20_B_ (3C, C_10_H_15_), 35.00_C_ (3C, C_10_H_15_), 28.82_D_ (3C, C_10_H_15_), 17.45 (CH_3_). HR-MS *m*/*z* 265.1374 [M^+^ + 1] (calcd for C_14_H_21_N_2_OS: 265.1375). R_f_ (silicagel, AcOEt:hexane 1:1): 0.38.

2-(adamantan-1-ylamino)-5-ethylthiazol-4(5*H*)-one (**3c**)—Yield: 66.1%. M.p. 257–259 °C. ^1^H-NMR (700 MHz, CDCl_3_, δ ppm, J Hz): 11.98 (br, 1H, NH), 4.24 (m, 1H, C^5^-H), 2.23 (s, 3H, Ad), 2.21–2.18 (m, 1H, C^5^-CH_A_), 2.17 (s, 6H, Ad), 2.10–2.02 (m, 1H, C^5^-CH_B_), 1.72 (dd, 6H, Ad, 12.7 29.6), 1.11 (dd, 3H, CH_3_, 7.0 7.7). ^13^C-NMR (100 Hz, CDCl_3_, δ ppm): 170.39 (C-4), 170.35 (C-2), 58.66_A_ (1C, C_10_H_15_), 51.90 (C-5), 40.15_B_ (3C, C_10_H_15_), 34.99_C_ (3C, C_10_H_15_), 28.82_D_ (3C, C_10_H_15_), 24.96 (CH_2_), 10.40 (CH_3_). HR-MS *m*/*z* 279.1536 [M^+^ + 1] (calcd for C_15_H_23_N_2_OS: 279.1531). R_f_ (silicagel, AcOEt:hexane 1:1): 0.46.

2-(adamantan-1-ylamino)-5-propylthiazol-4(5*H*)-one (**3d**)—Yield: 60.1%. M.p. 247–249 °C. ^1^H-NMR (700 MHz, CDCl_3_, δ ppm, J Hz): 11.91 (br, 1H, NH), 4.26 (s, 1H, C^5^-H), 2.27 (s, 3H, Ad), 2.21–2.18 (m, 1H, C^5^-CH_A_CH_2_CH_3_), 2.17 (s, 6H, Ad), 1.98–1.90 (m, 1H, C^5^-CH_B_CH_2_CH_3_), 1.72 (dd, 6H, Ad, 13.0 28.7), 1.56–1.46 (m, 2H, C^5^-CH_2_CH_2_-CH_3_), 1.01 (t, 3H, CH_3_, 7.4). ^13^C-NMR (100 Hz, CDCl_3_, δ ppm): 170.52 (C-4), 170.32 (C-2), 58.66_A_ (1C, C_10_H_15_), 50.43 (C-5), 40.16_B_ (3C, C_10_H_15_), 35.00_C_ (3C, C_10_H_15_), 33.53 (CH_2_), 28.82_D_ (3C, C_10_H_15_), 19.94 (CH_2_), 12.98 (CH_3_). HR-MS *m*/*z* 293.1696 [M^+^ + 1] (calcd for C_16_H_25_N_2_OS: 293.1688). R_f_ (silicagel, AcOEt:hexane 1:1): 0.53.

2-(adamantan-1-ylamino)-5-isopropylthiazol-4(5*H*)-one (**3e**)—Yield: 18.7%. M.p. 239–241 °C. ^1^H-NMR (700 MHz, CDCl_3_, δ ppm, J Hz): 5.46 (br, 1H, NH), 4.25 (d, 1H, C^5^-H 3.4), 2.61–2.55 (m, 1H C^5^-CH) 2.11 (s, 6H, Ad), 2.09 (s, 3H, Ad), 1.69 (dd, 6H, Ad, 11.6 23.2), 1.25 (s, 3H, CH_3_). ^13^C-NMR (100 Hz, CDCl_3_, δ ppm): 190.18 (C-4), 177.80 (C-2), 63.89_A_ (1C. C_10_H_15_), 56.49 (C-5), 41.07_B_ (3C, C_10_H_15_), 35.56_C_ (3C, C_10_H_15_), 30.32 (1C, CH(CH_3_)_2_), 29.30_D_ (1C, C_10_H_15_), 29.10_D_ (1C, C_10_H_15_), 28.97_D_ (1C, C_10_H_15_), 22.15 (1C, CH(CH_3B_)_2_), 15.78 (1C, CH(CH_3A_)_2_). HR-MS *m*/*z* 293.1697 [M^+^ + 1] (calcd for C_16_H_25_N_2_OS: 293.1688). R_f_ (silicagel, AcOEt:hexane 1:1): 0.50.

2-(adamantan-1-ylamino)-5,5-dimethylthiazol-4(5*H*)-one (**3f**)—Yield: 15.8%. M.p. 208–210 °C. ^1^H-NMR (700 MHz, CDCl_3_, δ ppm, J Hz): 5.47 (br, 1H, NH), 2.15 (s, 3H, Ad), 2.12 (s, 3H, Ad), 1.78 (s, 3H, Ad), 1.69 (dd, 6H, Ad, 10.8 24.2), 1.63 (s, 6H, 2xCH_3_). ^13^C-NMR (100 Hz, CDCl_3_, δ ppm): 176.12 (C-4), 100.62 (C-2), 61.11_A_ (1C, C_10_H_15_), 53.17 (C-5), 42.50_B_ (3C, C_10_H_15_), 35.72_C_ (3C, C_10_H_15_), 29.53_D_ (3C, C_10_H_15_), 17.45 (2C, CH(CH_3_)_2_). HR-MS *m*/*z* 279.1535 [M^+^ + 1] (calcd for C_15_H_23_N_2_OS: 279.1531). R_f_ (silicagel, AcOEt:hexane 1:1): 0.48.

2-(adamantan-1-ylamino)-5-phenylthiazol-4(5*H*)-one (**3g**)—Yield: 66.2%. M.p. 252–254 °C. ^1^H-NMR (700 MHz, CDCl_3_, δ ppm, J Hz): 7.37–7.28 (m, 5H, C_6_H_5_), 5.55 (br, 1H, NH), 5.22 (s, 1H, C^5^-H), 2.17 (s, 3H, Ad), 2.14 (s, 6H, Ad), 1.71 (dd, 6H, Ad, 11.7 26.6). ^13^C-NMR (100 Hz, CDCl_3_, δ ppm): 178.03 (C-4), 177.55 (C-2), 129.24 (1C_Ph_), 128.91 (2C_Ph_), 128.34 (1C_Ph_), 128.23 (2C_Ph_), 59.54_A_ (1C, C_10_H_15_), 57.26 (C-5), 41.54_B_ (3C, C_10_H_15_), 35.95_C_ (3C, C_10_H_15_), 29.54_D_ (3C, C_10_H_15_). HR-MS *m*/*z* 327.1532 [M^+^ + 1] (calcd for C_19_H_23_N_2_OS: 327.1531). R_f_ (silicagel, AcOEt:hexane 1:1): 0.53.

2-(adamantan-1-ylamino)-5-(4-bromophenyl)thiazol-4(5*H*)-one (**3h**)—Yield: 25.4%. M.p. 320 °C (dec.). ^1^H-NMR (700 MHz, DMSO, δ ppm, J Hz): 7.56–7.49 (dd, 2H, C_6_H_4_, 8.6 19.0), 7.33 (br, 1H, NH), 7.31–7.25 (d, 2H, C_6_H_4_, 7.0), 2.02 (s, 3H, Ad), 1.96 (dd, 6H, Ad, 13.0 26.0), 1.59 (s, 6H, Ad). HR-MS *m*/*z* 405.0639 [M^+^ + 1] (calcd for C_19_H_22_N_2_OS^79^Br: 405.0636). R_f_ (silicagel, AcOEt:hexane 1:1): 0.63.

2-(adamantan-1-ylamino)-1-thia-3-azaspiro [4.5]dec-2-en-4-one (**3i**)—Yield: 14.6%. M.p. 268–270 °C. ^1^H-NMR (700 MHz, DMSO, δ ppm, J Hz): 5.43 (s, 1H, NH), 2.12 (s, 6H, Ad), 2.05 (s, 3H, Ad), 1.99–1.75 (m, 6H, C_5_H_10_), 1.70 (dd, 6H, Ad, 13.7 24.6), 1.40–1.22 (m, 4H, C_5_H_10_). ^13^C-NMR (100 Hz, CDCl_3_, δ ppm): 193.39 (C-4), 177.15 (C-2), 70.66 (C-5), 56.79_A_ (1C, C_10_H_15_), 41.58_B_ (3C, C_10_H_15_), 36.73 (2C, C_5_H_10_), 35.97_C_ (3C, C_10_H_15_), 29.52_D_ (3C, C_10_H_15_), 25.50 (1C, C_5_H_10_), 24.86 (2C, C_5_H_10_). HR-MS *m*/*z* 319.1848 [M^+^ + 1] (calcd for C_18_H_27_N_2_OS: 319.1844). R_f_ (silicagel, AcOEt:hexane 1:1): 0.53.

6-(adamantan-1-ylamino)-5-thia-7-azaspiro[3.4]oct-6-en-8-one (**3j**)—Yield: 28.3%. M.p. 265–266 °C. ^1^H-NMR (700 MHz, DMSO, δ ppm, J Hz): 5.75 (s, 1H, NH), 2.86–2.76 (m, 2H, C_3_H_6_), 2.54–2.44 (m, 2H, C_3_H_6_), 2.33–2.23 (m, 1H, C_3_H_6_), 2.19–2.13 (m, 1H, C_3_H_6_), 2.10 (s, 6H, Ad), 2.04 (s, 3H, Ad), 1.70 (dd, 6H, Ad, 11.4 26.7). ^13^C-NMR (100 Hz, CDCl_3_, δ ppm): 193.19 (C-4), 175.71 (C-2), 61.52 (C-5), 56.37_A_ (1C, C_10_H_15_), 41.11_B_ (3C, C_10_H_15_), 35.56_C_ (3C, C_10_H_15_), 33.89 (2C, C_3_H_6_), 29.11_D_ (3C, C_10_H_15_), 16.52 (1C, C_3_H_6_). HR-MS *m*/*z* 291.1535 [M^+^ + 1] (calcd for C_16_H_23_N_2_OS: 291.1531). R_f_ (silicagel, AcOEt:hexane 1:1): 0.55.

### 3.4. Inhibition of 11β-HSD Assays 

#### 3.4.1. 11β-HSD1

Human liver microsomes were used as a source of 11β-HSD1 enzyme to study the inhibitory effect of **3a**–**3j** on the conversion of cortisone to cortisol [52]. Standard 96-well microplates were filled with reagent mixture: cortisone/NADPH (20 μL, to achieve the final concentration of 200 nM/2 μM), microsomes (10 μL, 1.13 μg of 11β-HSD1 in 1 mL) solution in PBS (final amount of 2.5 μg), phosphate buffer (60 μL, pH 7.4), and 10 µL of compounds **3a**–**3j** dissolved in the mixture containing 1% of DMSO and 99% of water. The resulting solution with a final volume of 100 µL was incubated for 2.5 h at 37 °C. To stop the reaction, 10 µL of a 100 µM solution of 18β-glycyrrhetinic acid in PBS was added. The level of cortisol obtained in the reaction was measured by commercially available ELISA kit.

#### 3.4.2. 11β-HSD2

Human liver microsomes were used as a source of 11β-HSD2 enzyme to study the inhibitory effect of **3a**–**3j** on the conversion of cortisol to cortisone. Standard 96-well microplates were filled with reagent mixture: cortisol/NAD^+^ (20 μL, to achieve the final concentration of 200 nM/2 μM), microsomes (10 μL, 0.127 μg of 11β-HSD2 in 1 mL) solution in PBS (final amount of 2.5 μg), phosphate buffer (60 μL, pH 7.4), and 10 µL of compounds **3a**–**3j** dissolved in the mixture containing 1% of DMSO and 99% of water. The resulting solution with the final volume of 100 µL was incubated for 2.5 h at 37 °C. To stop the reaction, 10 μL of a 100 μM solution of carbenoxolone in PBS was added. The level of unreacted cortisol was measured by commercially available ELISA kit.

#### 3.4.3. Determination of IC_50_

Calibration curves to determine IC_50_ values for compounds **3a**–**3j** were obtained using their solutions at concentrations of 0.625, 1.25, 2.5, 5.0, and 10.0 µM and using standard procedure and conditions as described in the previous sections. As a control, analogous tests without the addition of inhibitors were used. Half the inhibitory concentration (causing 50% reduction of cortisol or cortisone) was read from the chart.

### 3.5. Molecular Docking

Ligand molecules (see Table 1) were drawn manually by using the Avogadro 1.1.1 software [53] and optimized within the UFF force field [54] (5000 steps, steepest descent algorithm). Six ligands are chiral compounds; in their cases, docking was performed separately for each stereoisomer. The flexible, optimized ligand molecules were docked into the binding pocket of the eight following protein structures found in the PDB database: 3crz, 3g49, 3qqp, 4bb5, 4c7j, 4hfr, 4p38, and 4yyz. The AutoDock Vina software [55] was applied for docking simulations. The procedure of docking was carried out within the cuboid region of dimensions of 18 × 18 × 18 Å^3^ which covers the originally co-crystallized ligands present in the PDB structures as well as the closest amino-acid residues that exhibit contact with those ligands. All the default procedures and algorithms implemented in AutoDock Vina were applied during the docking procedure. In addition to the flexibility of the ligand molecules, the rotation of selected sidechains (Leu126, Leu171, Tyr177, Tyr183, Leu215, Leu217) in the proximity of the co-crystalized ligands was allowed. The predicted binding energies were averaged over all the eight protein structures. In addition, in the case of chiral ligands, the energies were averaged over two stereoisomers. The more favorable binding mode is associated with the lower binding energy value; only the lowest energy values and ligand poses associated with them were considered in the subsequent analysis.

The docking methodology was initially validated by docking simulations of the ligand molecule originally included in the protein structure. The description of the validation procedure and the graphical illustration of its results can be found in [30]. Moreover, the same docking procedure was applied in order to recover the poses of all ligands originally present in the above-mentioned eight PDB entries. In all cases, the accepted methodology appeared to be accurate enough to recover the original positions of the bound ligands.

## 4. Conclusions

In conclusion, we obtained 10 new 2-(adamantan-1-ylamino)thiazol-4(5*H*)-one derivatives containing different substituents at C-5 of thiazole ring and tested their activity towards inhibition of two 11β-HSD isoforms. All the obtained compounds show inhibitory activity against both isoforms to a different extent (15.3–82.8% for 11β-HSD1 and 14.4–44.7% for 11β-HSD2).

The binding energies found during docking simulations for 11β-HSD1 correctly reproduces the experimental IC_50_ values for analyzed compounds. Molecular docking shows that the driving force for binding between ligands and protein related to the hydrophobic interactions of the ligand with Tyr177 and neighboring leucines, supported by the π–π interactions with Tyr183 and NADP^+^, whereas divergences between binding energies across the whole group of compounds can be ascribed to the interactions between the substituent attached to the thiazole ring and the region of the protein located in the vicinity of Thr124 and Thr222.

The most active compound **3i** at the concentration of 10 µM inhibits the activity of isoform 1 by 82.82%. This value is comparable to the known inhibitor-carbenoxolone. The IC_50_ value is twice the value determined by us for carbenoxolone, however, inhibition of the enzyme isoform 2 to a lesser extent makes it an excellent material for further tests.

## Figures and Tables

**Table 1:**
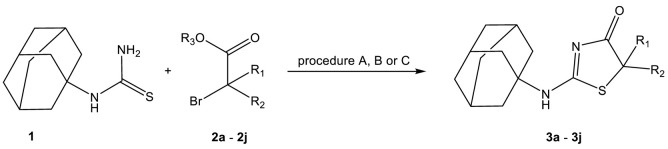
Synthesis of 2-(adamantan-1-ylamino)thiazol-4(5*H*)-one.

No.	R_1_	R_2_	Procedure	Isolated Yield [%]	M.p. (°C)
**3a**	H	H	A	64.1	271–273
**3b**	H	CH_3_	A	75.8	265–267
**3c**	H	C_2_H_5_	A	66.1	257–259
**3d**	H	C_3_H_7_	A	60.1	247–249
**3e**	H	CH(CH_3_)_2_	B	18.7	239–241
**3f**	CH_3_	CH_3_	B	15.8	208–210
**3g**	H	C_6_H_5_	A	66.2	252–254
**3h**	H	C_6_H_5_*p*-Br	A	25.4	320 (dec.)
**3i**	-(CH_2_)_5_-		C	14.6	268–270
**3j**	-(CH_2_)_3_-		C	28.3	265–266

A-MeOH, MeONa; reflux, B: CHCl_3_, RT; C: EtOH, DIPEA, reflux.

**Table 2:** Inhibitory activity of 2-(adamantan-1-ylamino)thiazol-4(5*H*)-one derivatives. The comparison of the experimental data and the binding energies collected in the docking study. The calculation results were averaged over 8 protein structures (11β-HSD1) available in the PDB database; the corresponding standard deviations are given.

No.	R_1_	R_2_	% of 11β-HSD1 Inhibition 10 μM	IC_50_ 11β-HSD1 [µM]	Binding Energy [kcal/mol]	% of 11β-HSD2 Inhibition 10 μM
**3a**	H	H	22.27 ± 5.31	nd	−7.91 ± 0.20	29.81 ± 4.21
**3b**	H	CH_3_	62.15 ± 3.59	3.52 ± 0.19	−8.48 ± 0.23 ^c^	44.71 ± 4.19
**3c**	H	C_2_H_5_	69.22 ± 5.12	5.44 ± 0.32	−8.58 ± 0.24 ^c^	14.42 ± 1.16
**3d**	H	C_3_H_7_	72.37 ± 2.63	3.23 ± 0.26	−8.63 ± 0.27 ^c^	28.85 ± 4.11
**3e**	H	CH(CH_3_)_2_	76.40 ± 2.55	1.19 ± 0.21	−8.96 ± 0.31 ^c^	16.35 ± 2.06
**3f**	CH_3_	CH_3_	65.99 ± 1.12	6.11 ± 0.62	−8.46 ± 0.11	24.52 ± 3.24
**3g**	H	C_6_H_5_	46.32 ± 5.53	nd	−9.99 ± 0.45 ^c^	18.27 ± 3.18
**3h**	H	C_6_H_5_*p*-Br	15.30 ± 0.49	nd	−10.14 ± 0.46 ^c^	41.83 ± 6.12
**3i**	-(CH_2_)_5_-		82.82 ± 2.05	0.31 ± 0.05	−10.04 ± 0.33	44.71 ± 3.37
**3j**	-(CH_2_)_3_-		74.13 ± 2.85	3.32 ± 0.24	−8.98 ± 0.16	41.35 ± 4.22
control	-		84.78 ± 6.23 ^a^	0.16 ± 0.15 ^a^	-	46.15 ± 3.16 ^b^/ 55.77 ± 4.28 ^a^

^a^ for carbenoxolone, ^b^ for 11β-glycyrrhetinic acid, ^c^ value additionally averaged over two stereoisomers of the given ligand.

**Table 3:** Physicochemical properties of the obtained series of 2-(adamantylamino)thiazol-4(5*H*)-one derivatives.

Compound	miLogP	tPSA [A^2^]	Molecular Weight [g/mol]	nON	nOHNH	Number Rotatable Bonds	PAINS *
**3a**	2.64	41.46	250.37	3	1	2	0
**3b**	3.00	41.46	264.39	3	1	2	0
**3c**	3.51	41.46	278.42	3	1	3	0
**3d**	4.07	41.46	292.45	3	1	4	0
**3e**	3.75	41.46	292.45	3	1	3	0
**3f**	3.45	41.46	278.42	3	1	2	0
**3g**	4.22	41.46	326.46	3	1	3	0
**3h**	5.03	41.46	405.36	3	1	3	0
**3i**	4.62	41.46	318.49	3	1	2	0
**3j**	3.37	41.46	290.43	3	1	2	0

nOHNH—hydrogen bond donor; nON—hydrogen bond acceptor, tPSA—topological polar surface area, * Alerts of PAINS were determined according [47,48,49].

## Data Availability

Data available from the authors.

## Supplementary Materials

The following are available online at https://www.mdpi.com/article/10.3390/ijms22168609/s1. Figure S1A: ^1^H NMR spectra of compound **3a**, Figure S1B: ^1^H NMR spectra of compound **3b**, Figure S1C: ^1^H NMR spectra of compound **3c**, Figure S1D: ^1^H NMR spectra of compound **3d**, Figure S1E: ^1^H NMR spectra of compound **3e**, Figure S1F: ^1^H NMR spectra of compound **3f**, Figure S1G: ^1^H NMR spectra of compound **3g**, Figure S1H: ^1^H NMR spectra of compound **3h**, Figure S1I: ^1^H NMR spectra of compound **3i**, Figure S1J: ^1^H NMR spectra of compound **3j**, Figure S2A: ^13^C NMR spectra of compound **3a**, Figure S2B: ^13^C NMR spectra of compound **3b**, Figure S2C: ^13^C NMR spectra of compound **3c**, Figure S2D: ^13^C NMR spectra of compound **3d**, Figure S2E: ^13^C NMR spectra of compound **3e**, Figure S2F: ^13^C NMR spectra of compound **3f**, Figure S2G: ^13^C NMR spectra of compound **3g**, Figure S2H: ^13^C NMR spectra of compound **3i**, Figure S2I: ^13^C NMR spectra of compound **3j**, Figure S3A: Mass spectrum of compound **3a**, Figure S3B: Mass spectrum of compound **3b**, Figure S3C: Mass spectrum of compound **3c**, Figure S3D: Mass spectrum of compound **3d**, Figure S3E: Mass spectrum of compound **3e**, Figure S3F: Mass spectrum of compound **3f**, Figure S3G: Mass spectrum of compound **3g**, Figure S3H: Mass spectrum of compound **3h**, Figure S3I: Mass spectrum of compound **3i**, Figure S3J: Mass spectrum of compound **3j**.
