# Supplementary material for: Novel 2-(Adamantan-1-ylamino)Thiazol-4(5H)-One Derivatives and Their Inhibitory Activity towards 11β-HSD1—Synthesis, Molecular Docking and In Vitro Studies
Source: Int J Mol Sci. 2021 Aug 10;22(16):8609. doi: 10.3390/ijms22168609 (PMC8395285; doi:10.3390/ijms22168609)
Supplement: Supplementary file 1 [file ijms-22-08609-s001.zip › ijms-1296370-supplementary.pdf]

International Journal of *Molecular Sciences*

MDPI

Novel 2-(Adamantan-1-ylamino)Thiazol-4(5*H*)-One Derivatives and Their Inhibitory Activity towards 11β-HSD1 – synthesis, Molecular Docking and In Vitro Studies

Renata Studzińska [1,*], Daria Kupczyk [2], Wojciech Płaziński [3], Szymon Baumgart [1], Rafał Bilski [2], Renata Paprocka [1] and Renata Kołodziejska [2]

[1] Department of Organic Chemistry, Faculty of Pharmacy, Collegium Medicum in Bydgoszcz, Nicolaus Copernicus University in Toruń, 2 Jurasza Str., 85–089 Bydgoszcz, Poland; sz.baumgart@cm.umk.pl (Sz.B.); renata.bursa@cm.umk.pl (R.P.)

[2] Department of Medical Biology and Biochemistry, Faculty of Medicine, Collegium Medicum in Bydgoszcz, Nicolaus Copernicus University in Toruń, 24 Karłowicza Str., 85–092 Bydgoszcz, Poland; dariak@cm.umk.pl (D.K.); rafal.bilski@cm.umk.pl (R.B.); renatak@cm.umk.pl (R.K.)

[3] J. Haber Institute of Catalysis and Surface Chemistry, Polish Academy of Sciences, 8 Niezapominajek Str., 30-239 Cracow, Poland; wojtek_plazinski@tlen.pl

* Correspondence: rstud@cm.umk.pl

Supplementary information

^{1}H NMR spectra of compounds **3a–j.**

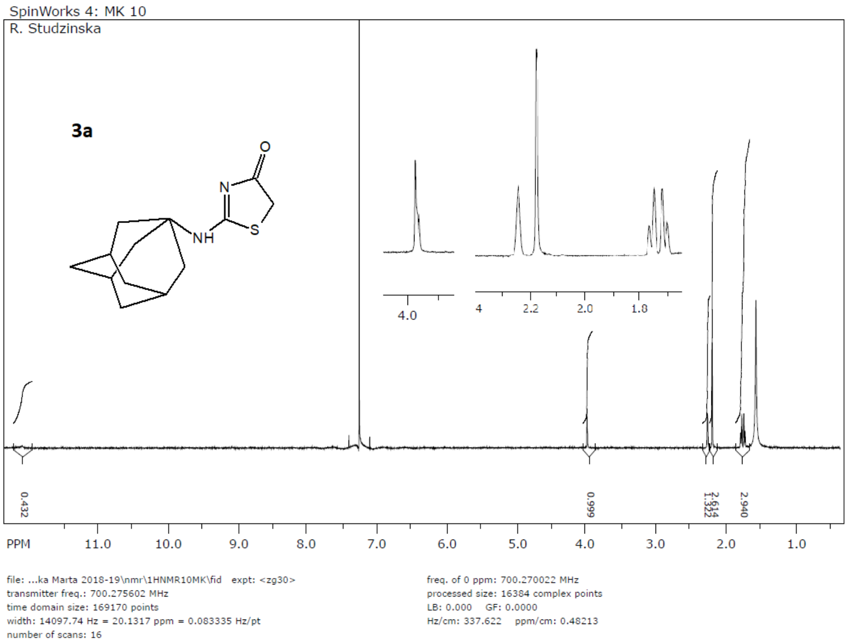

Figure S1A ^{1}H NMR spectra of compound **3a.**

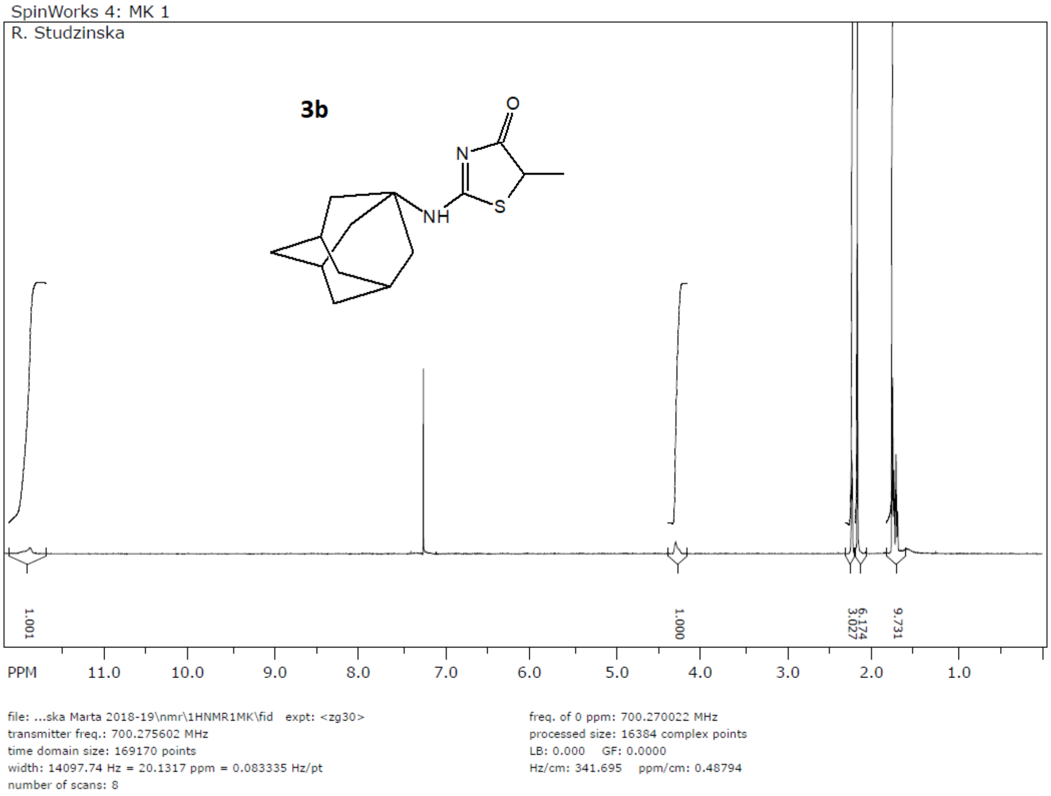

Figure S1B ^{1}H NMR spectra of compound **3b.**

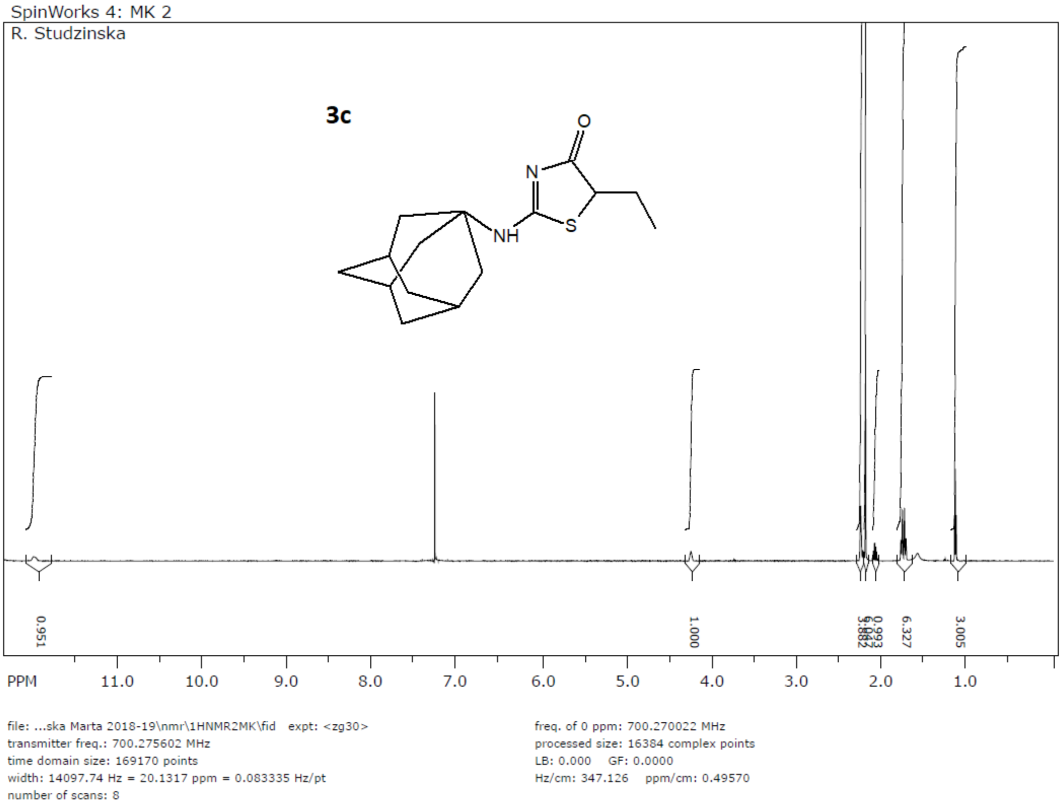

Figure S1C. ^{1}H NMR spectra of compound **3c.**

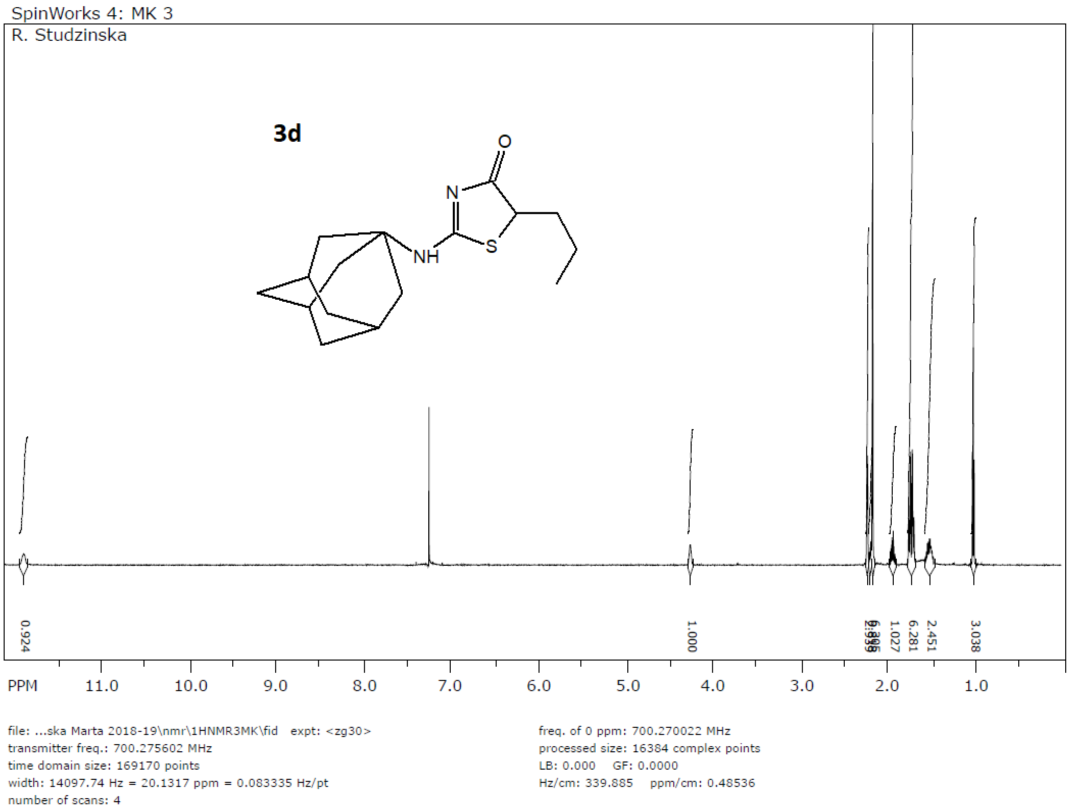

Figure S1D. ^{1}H NMR spectra of compound **3d.**

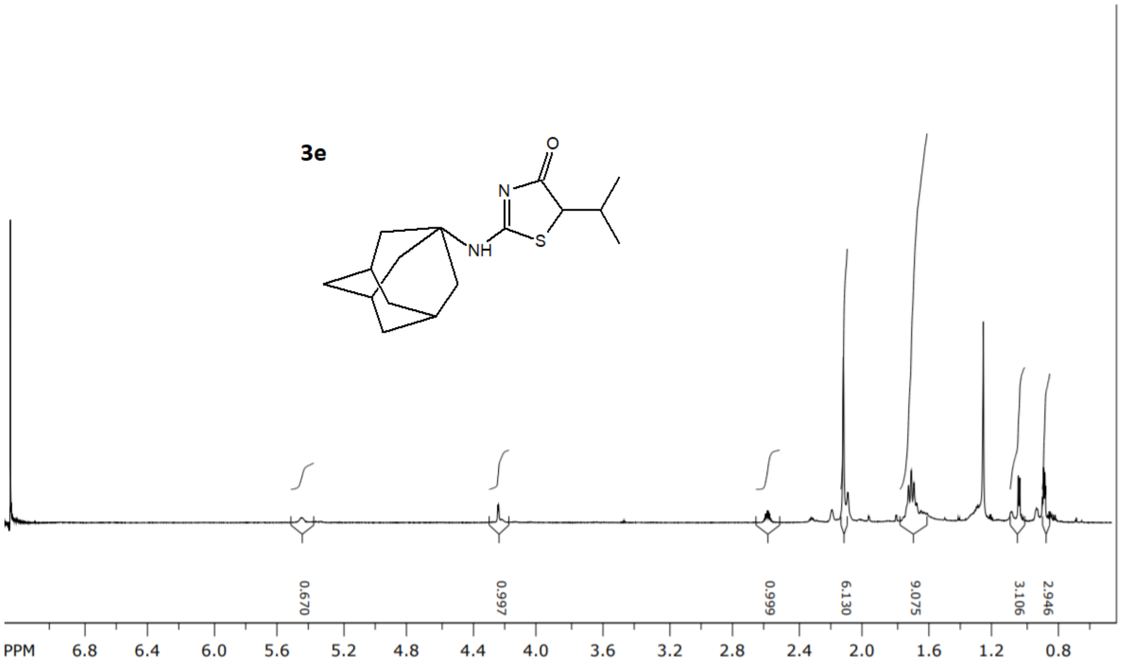

Figure S1E. ^{1}H NMR spectra of compounds **3e.**

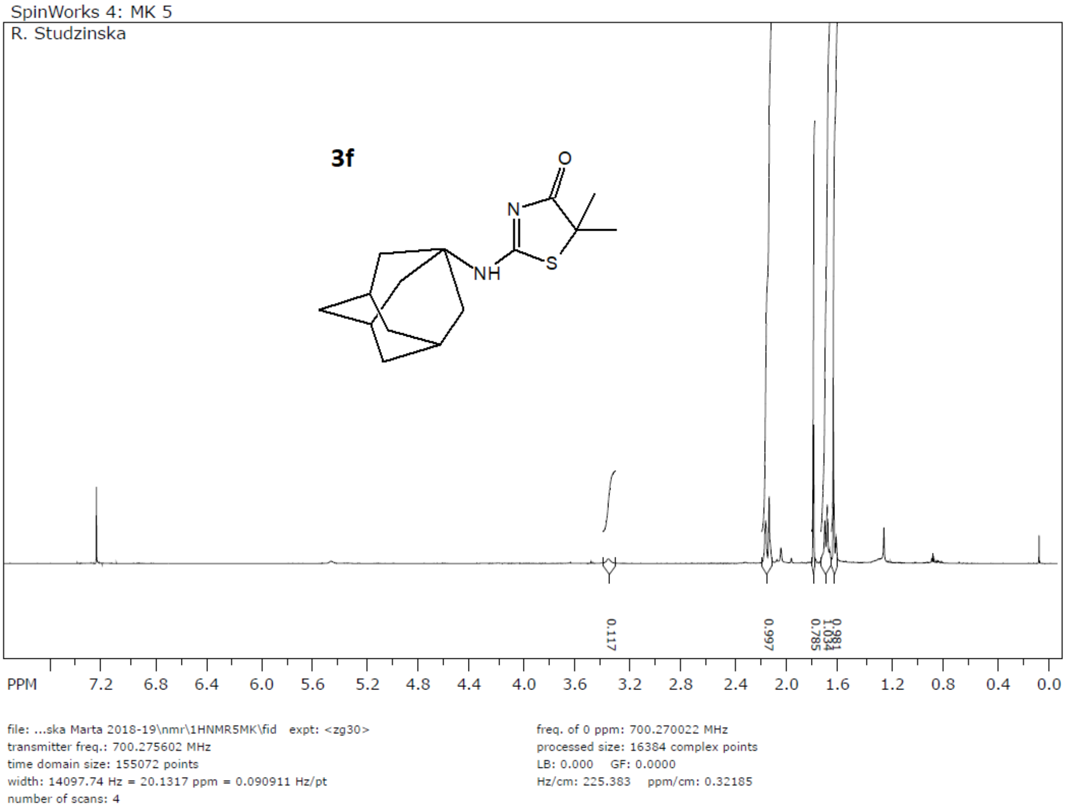

Figure S1F. ^{1}H NMR spectra of compound **3f.**

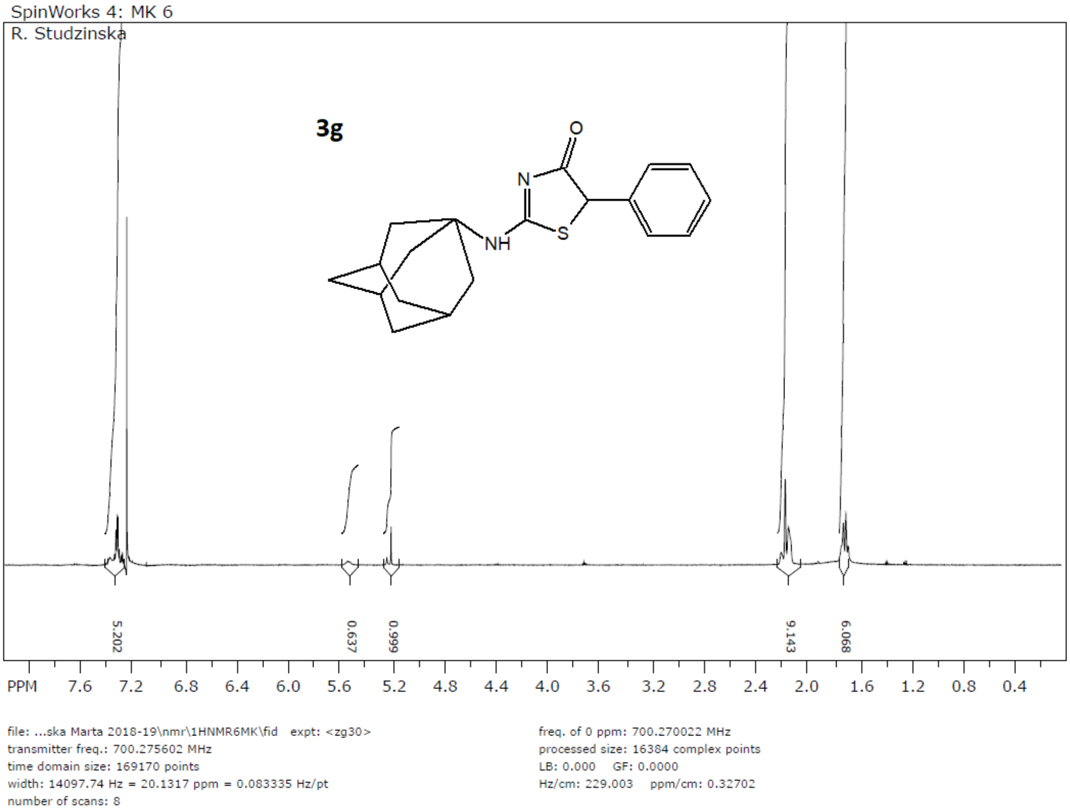

Figure S1G. ^{1}H NMR spectra of compound **3g.**

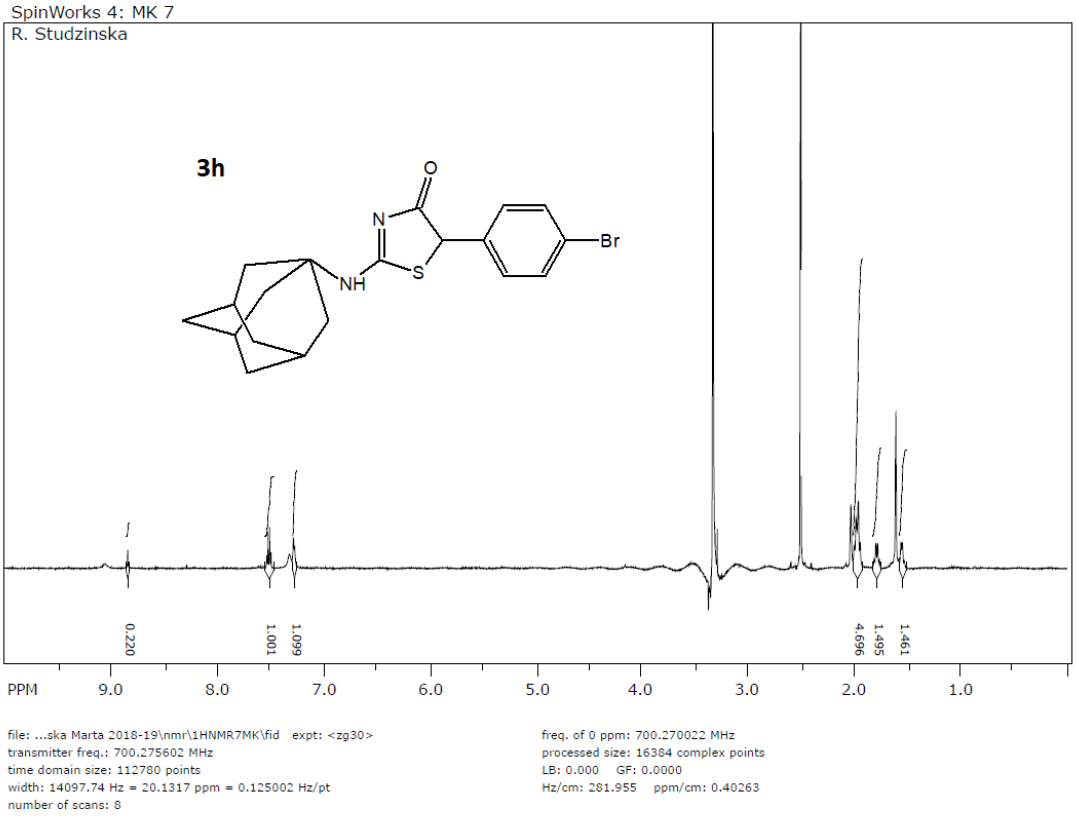

Figure S1H. ¹H NMR spectra of compound **3h.**

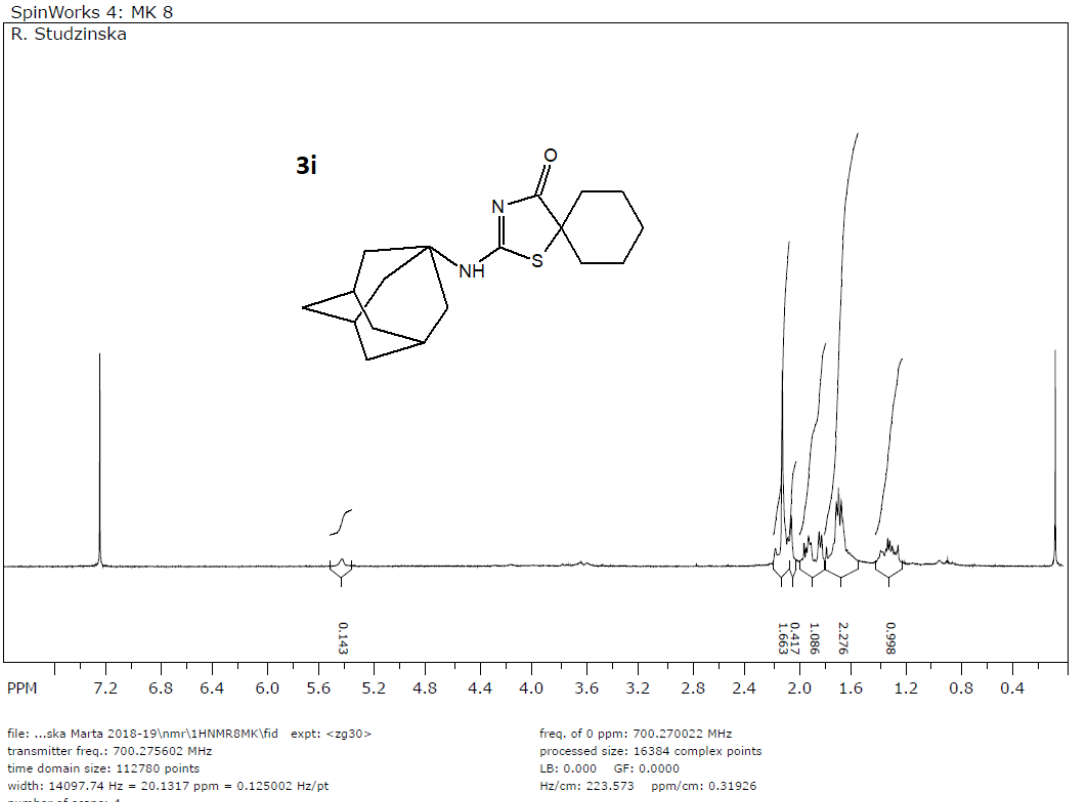

Figure S1I. ¹H NMR spectra of compound **3i.**

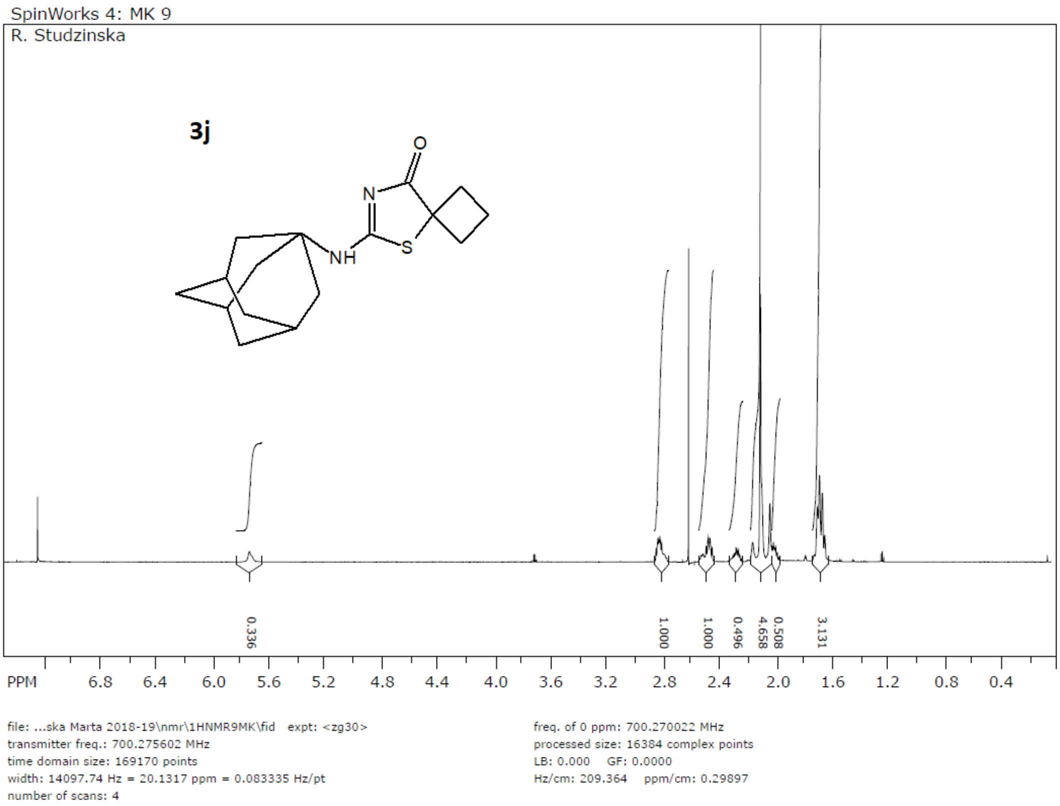

Figure S1J. ^{1}H NMR spectra of compound **3j.**

^{13}C NMR spectra of compounds **3a–j**

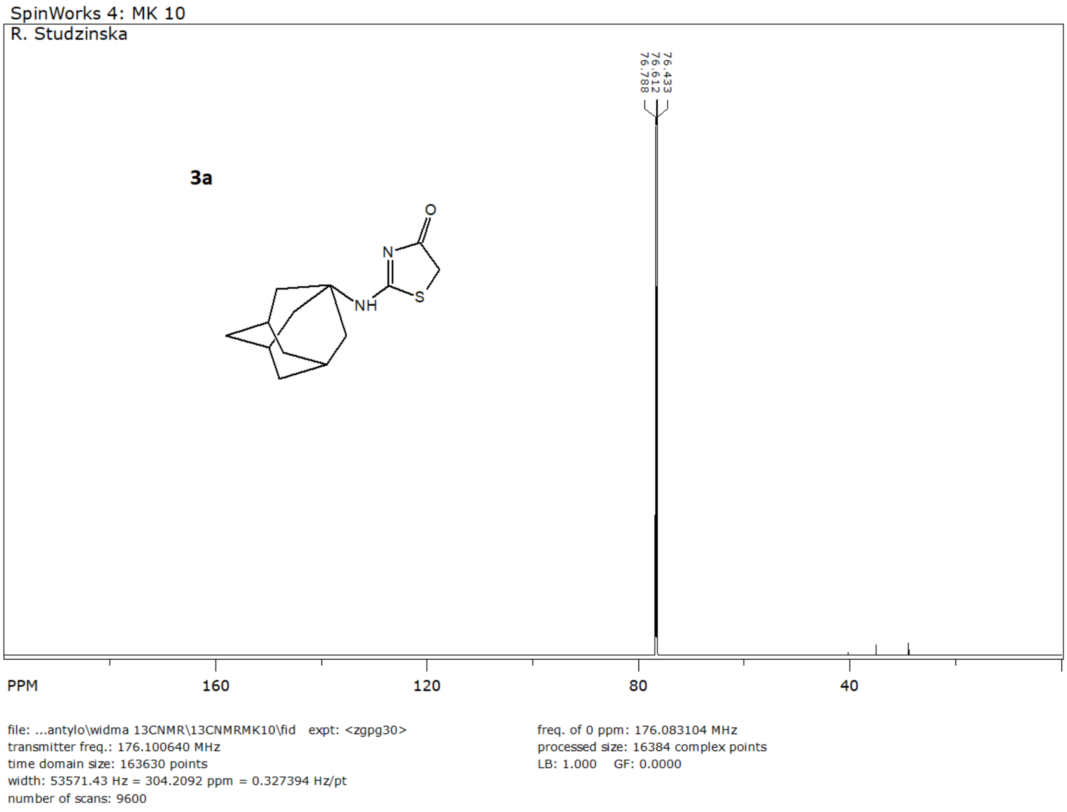

Figure S2A. ^{13}C NMR spectra of compound **3a.**

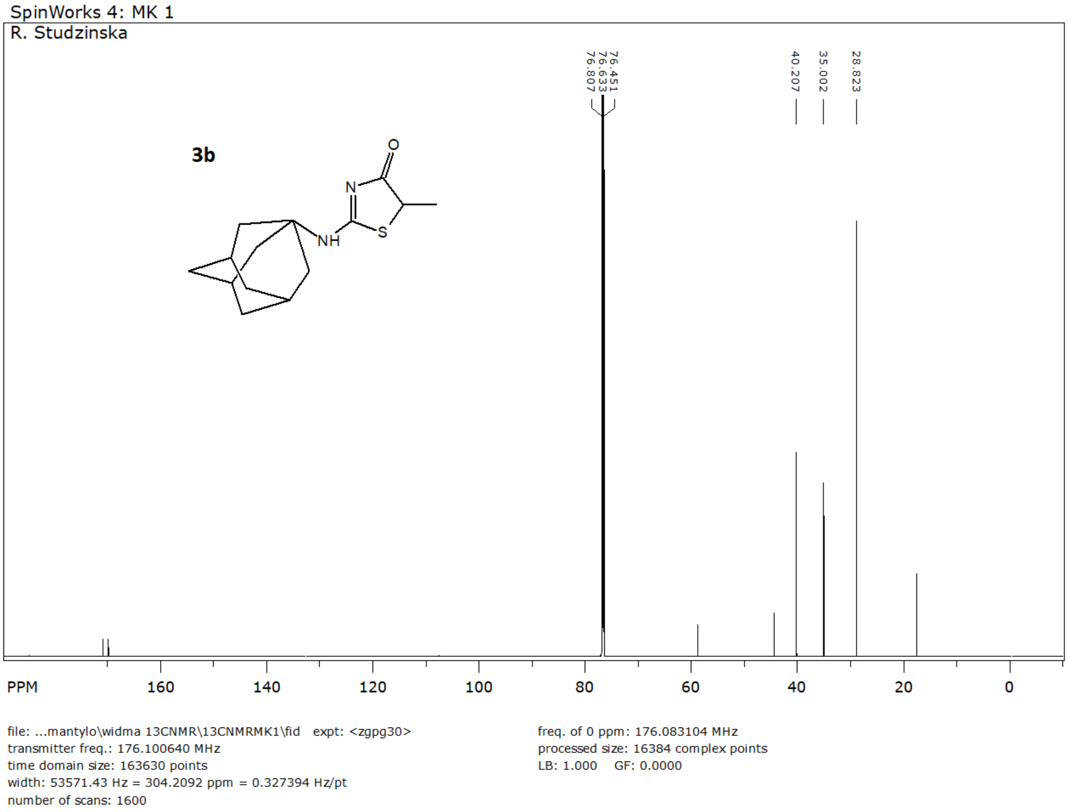

Figure S2B. ^{13}C NMR spectra of compound **3b.**

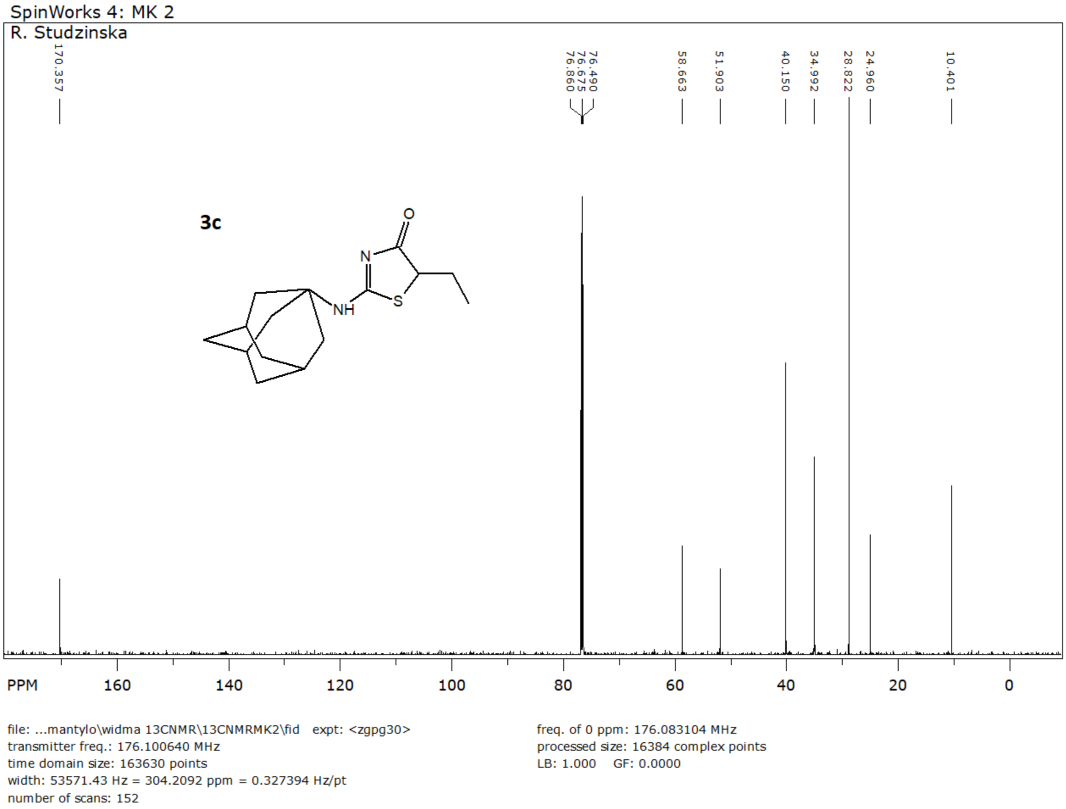

Figure S2C. ^{13}C NMR spectra of compound **3c.**

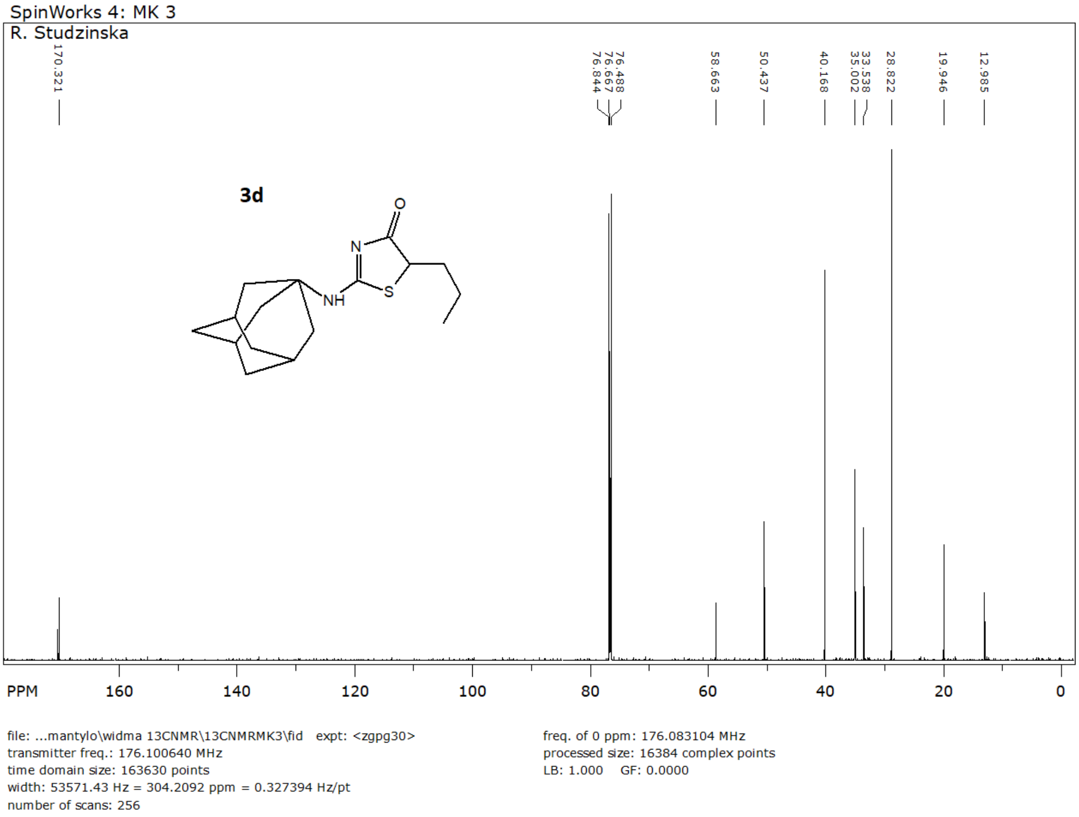

Figure S2D. ^{13}C NMR spectra of compound **3d.**

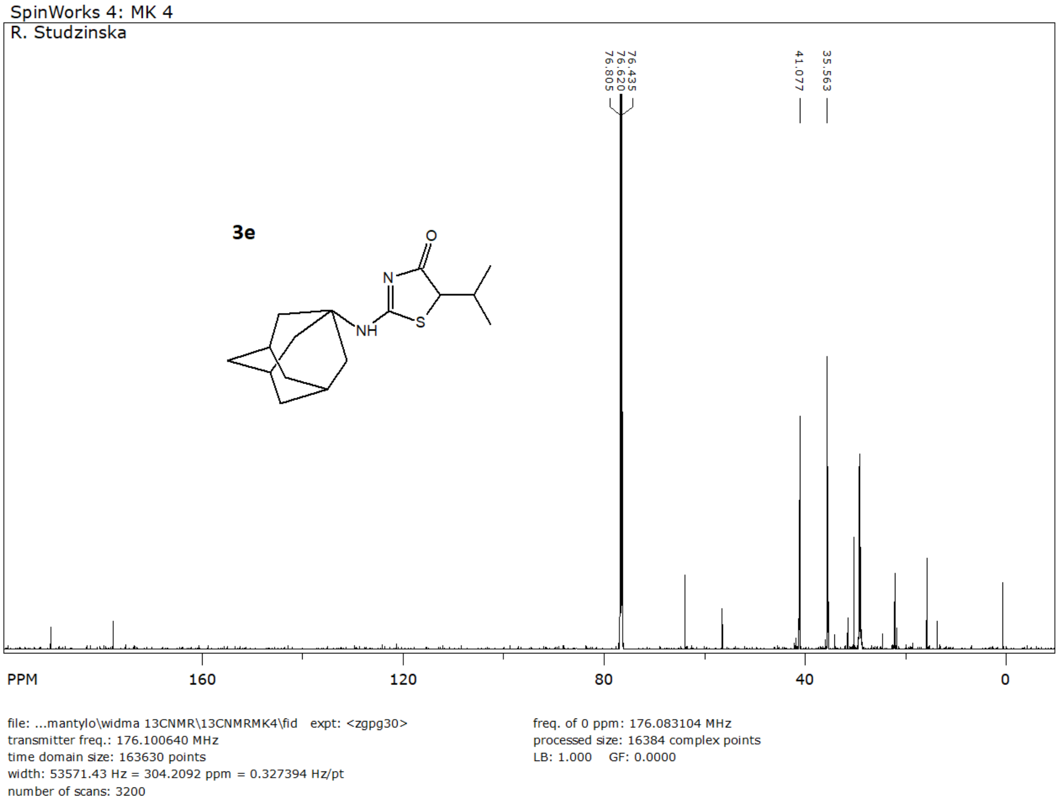

Figure S2E. ^{13}C NMR spectra of compound **3e.**

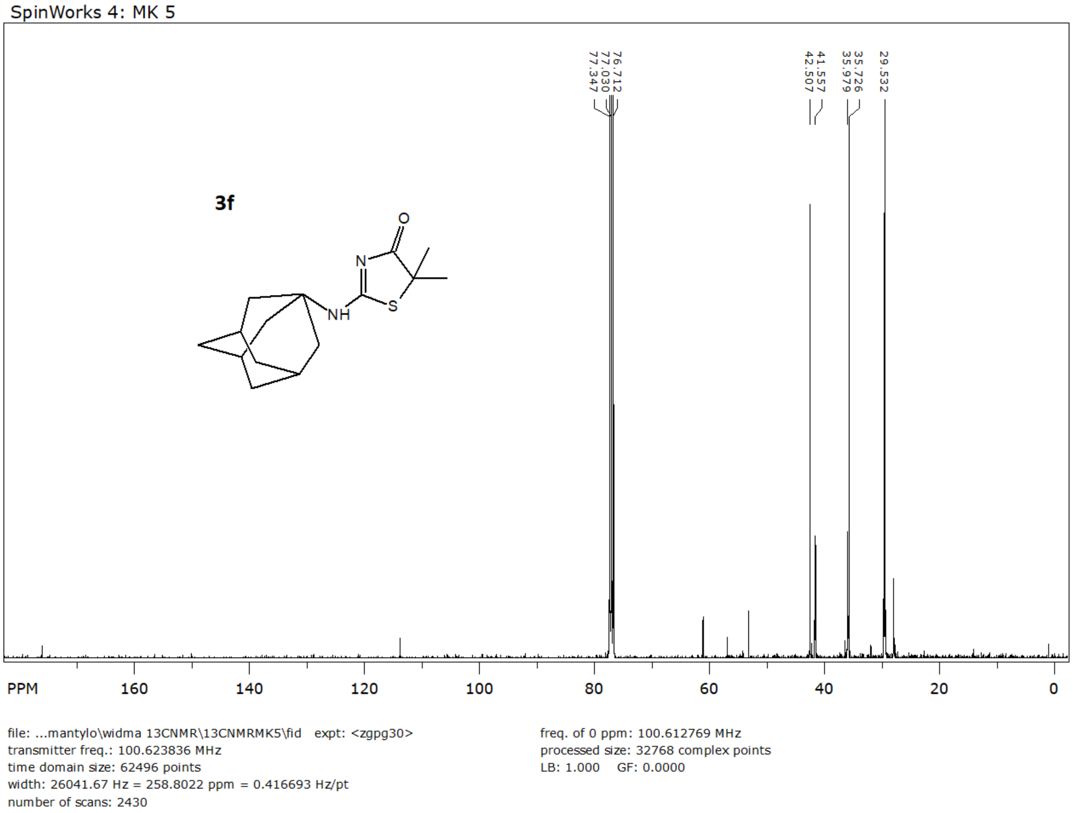

Figure S2F. ^{13}C NMR spectra of compound **3f.**

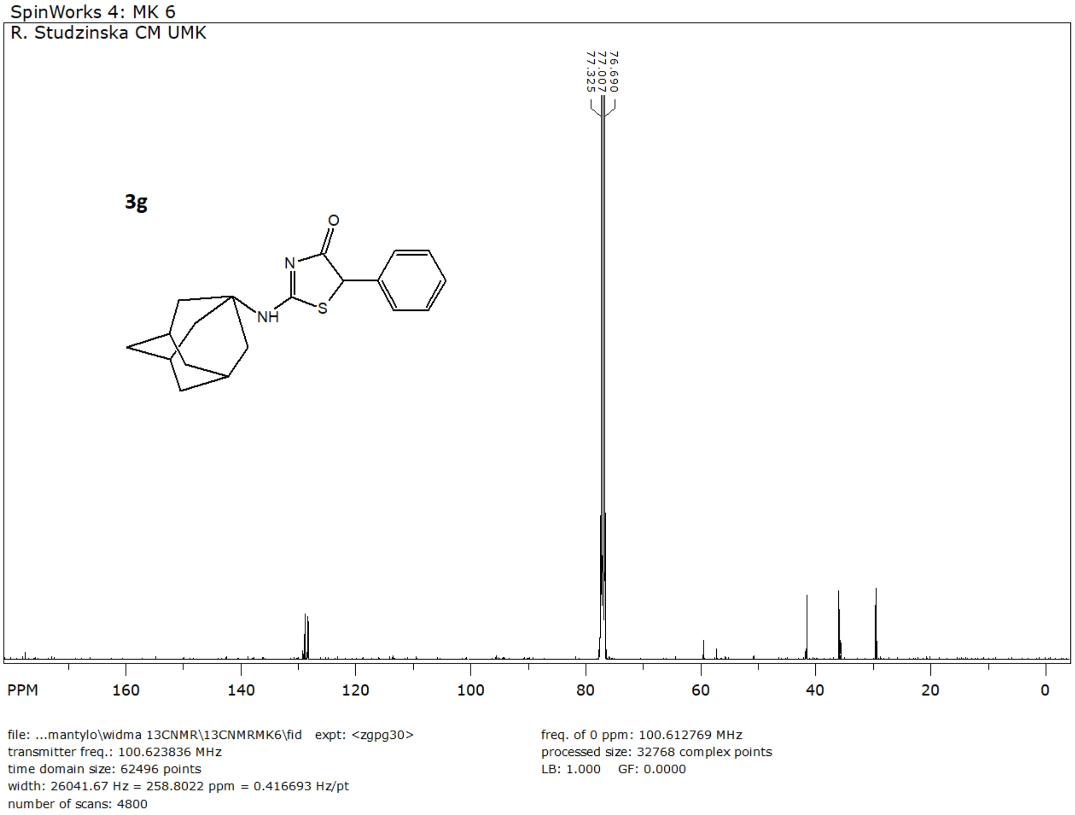

Figure S2G. ^{13}C NMR spectra of compound **3g.**

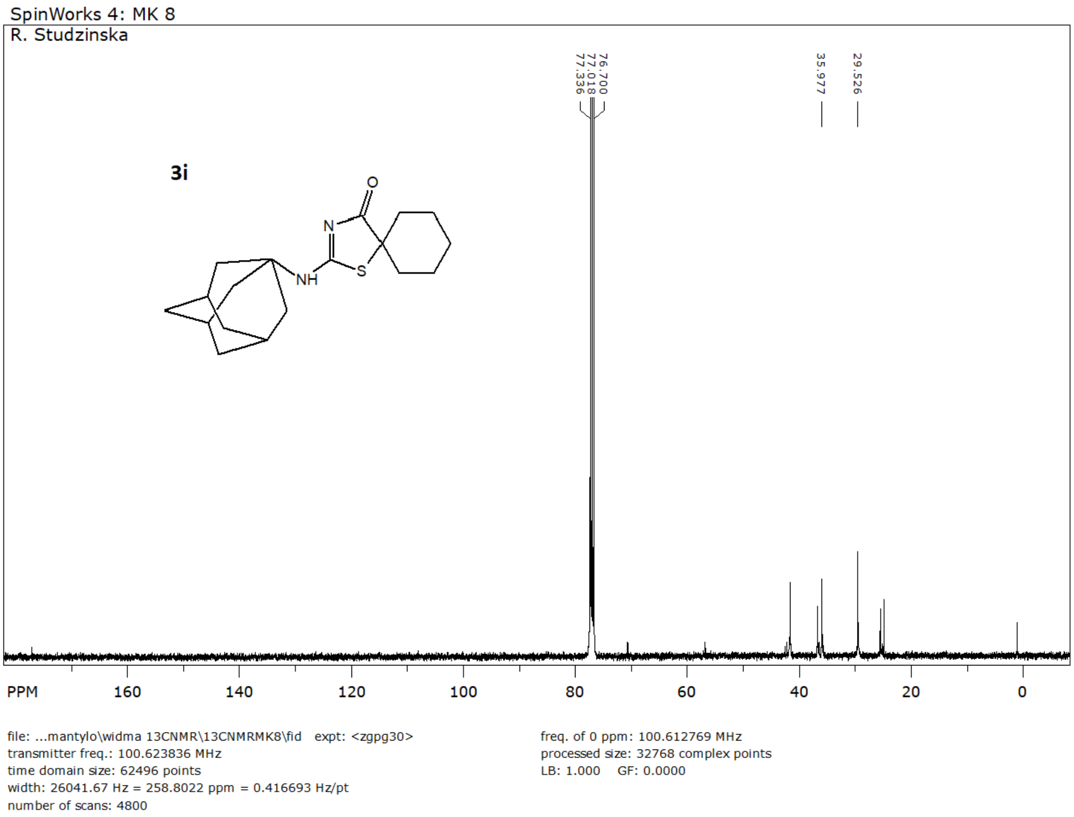

Figure S2H. ^{13}C NMR spectra of compound **3i.**

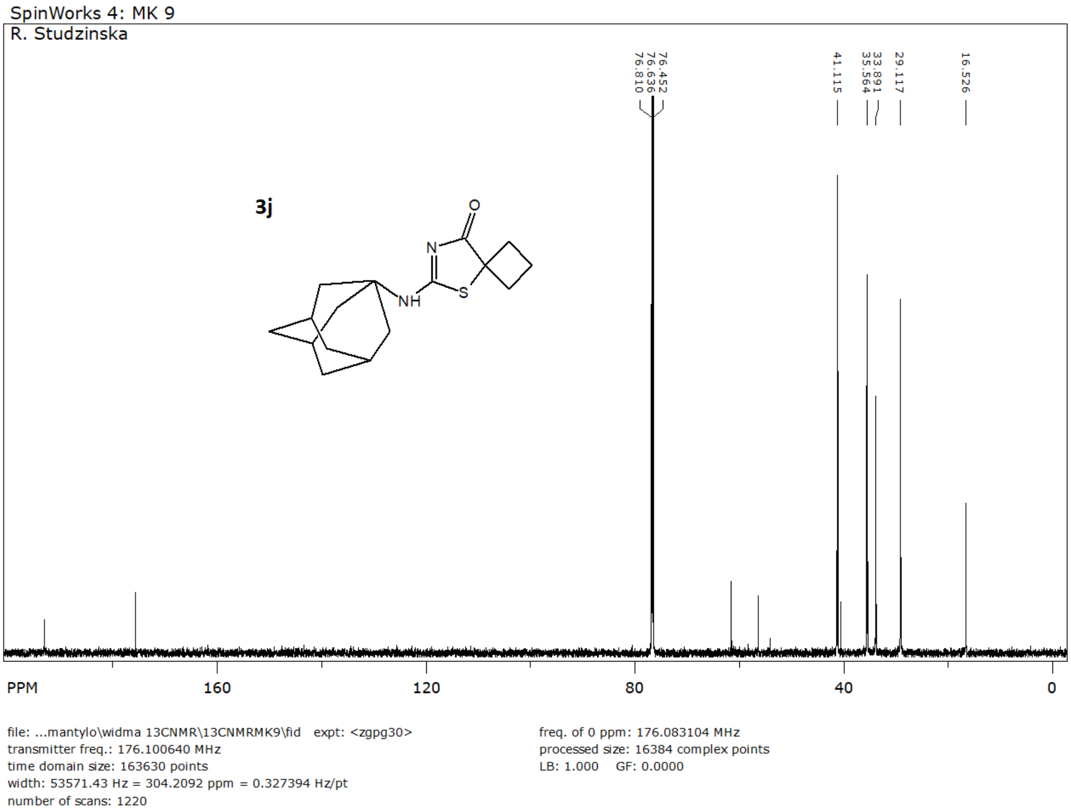

Figure S2I. ^{13}C NMR spectra of compound **3j.**

Mass spectrum of compounds **3a–j**

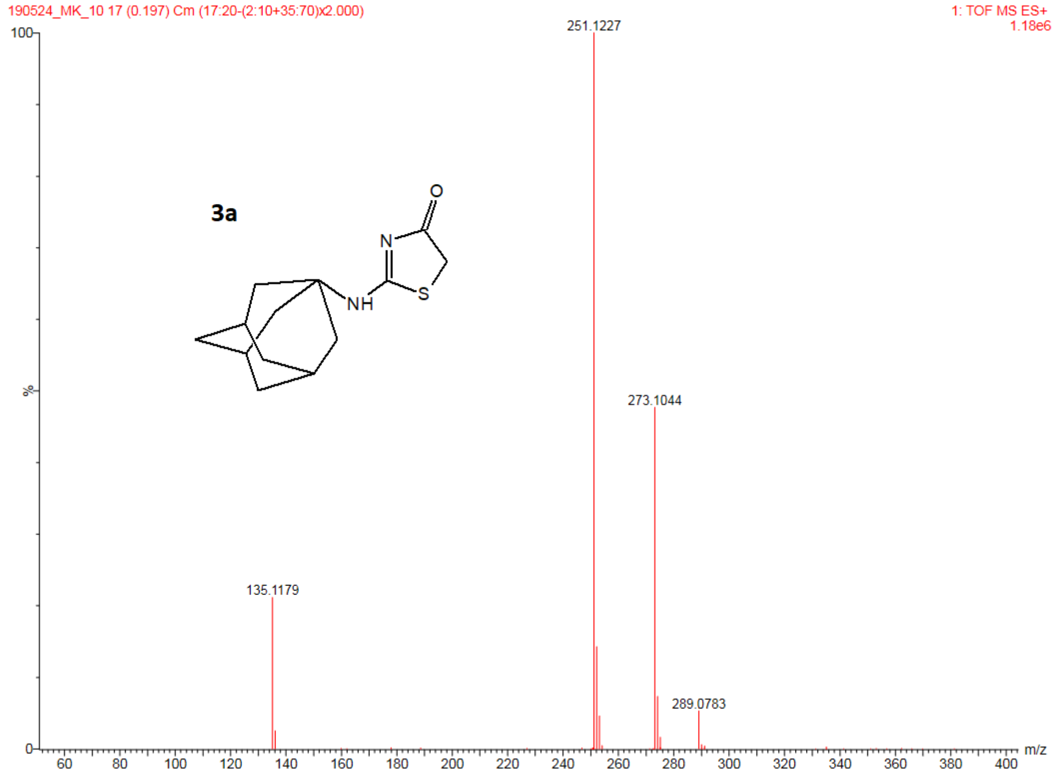

Figure S3A. Mass spectrum of compound **3a.**

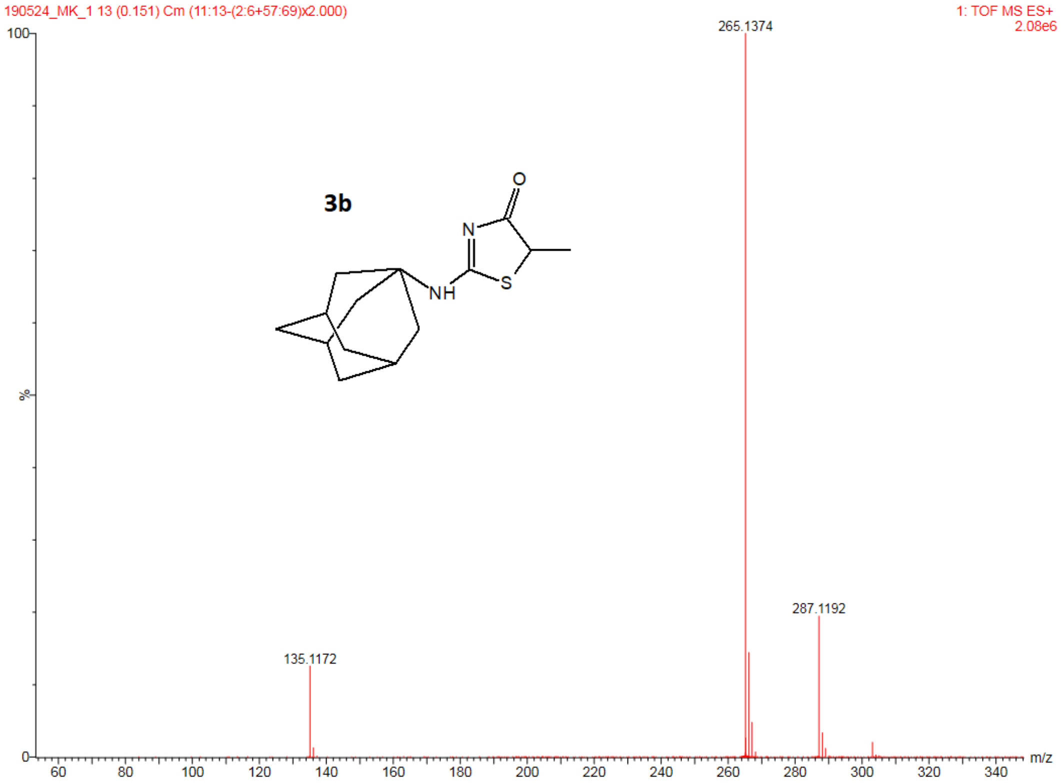

Figure S3B. Mass spectrum of compound **3b.**

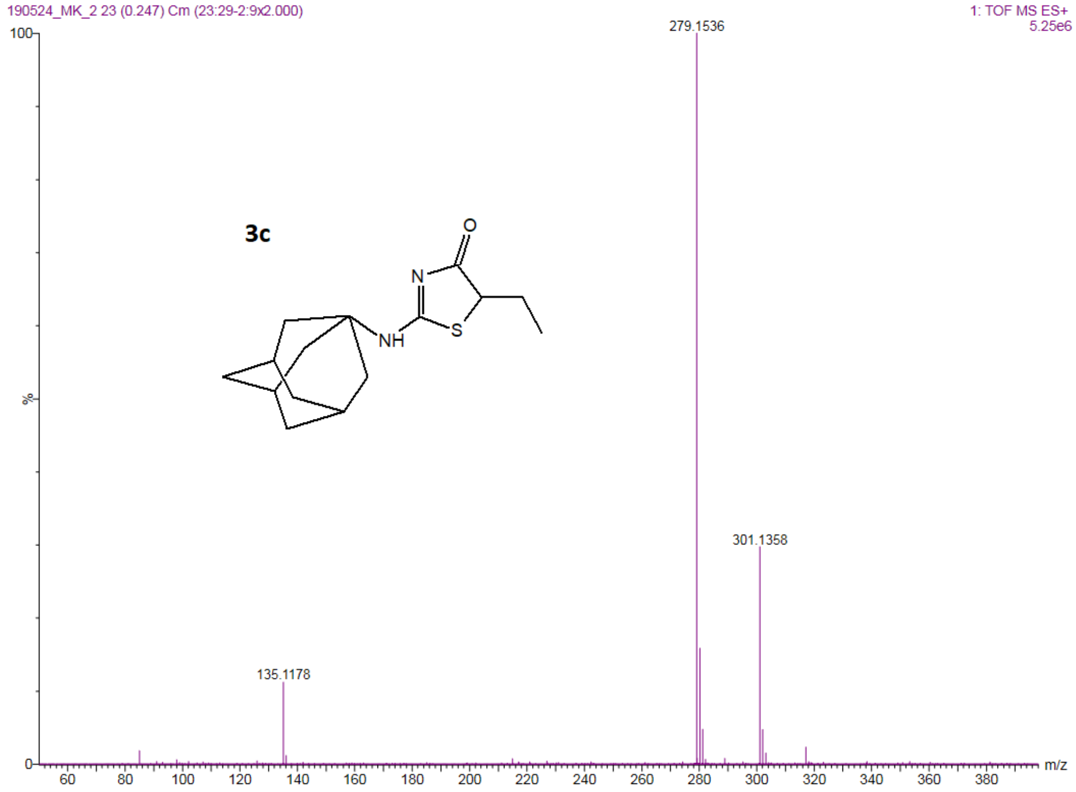

Figure S3C. Mass spectrum of compound **3c.**

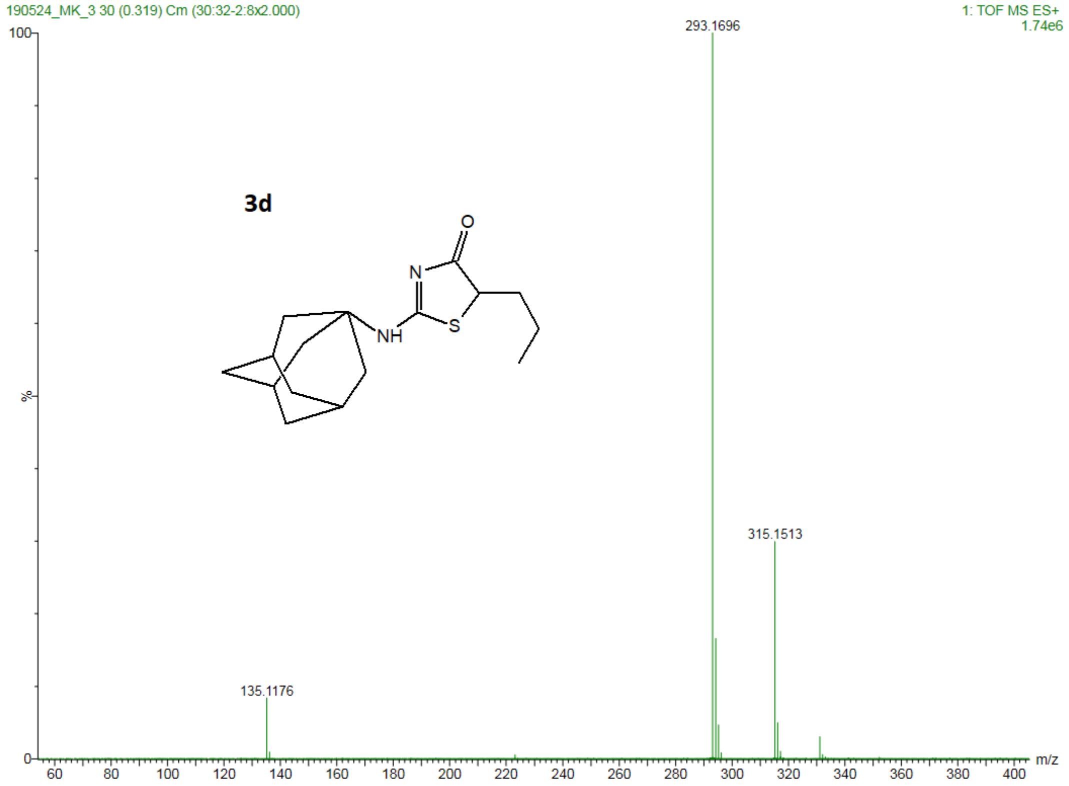

Figure S3D. Mass spectrum of compound **3d.**

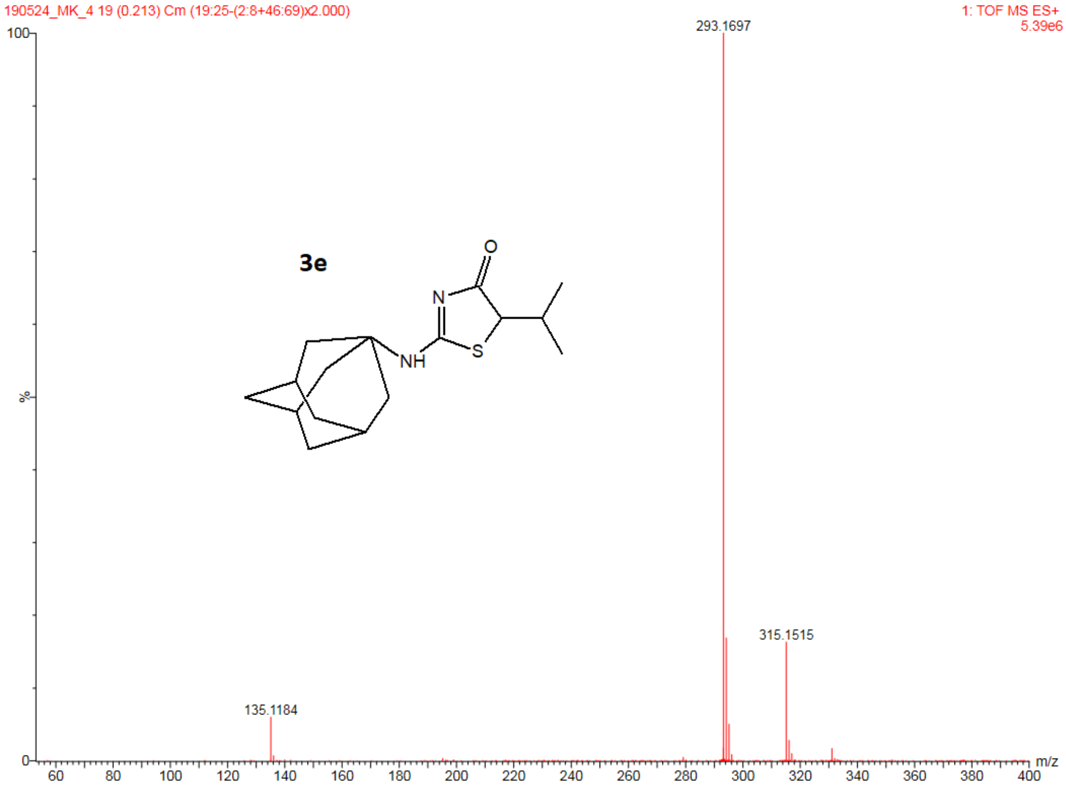

Figure S3E. Mass spectrum of compound **3e.**

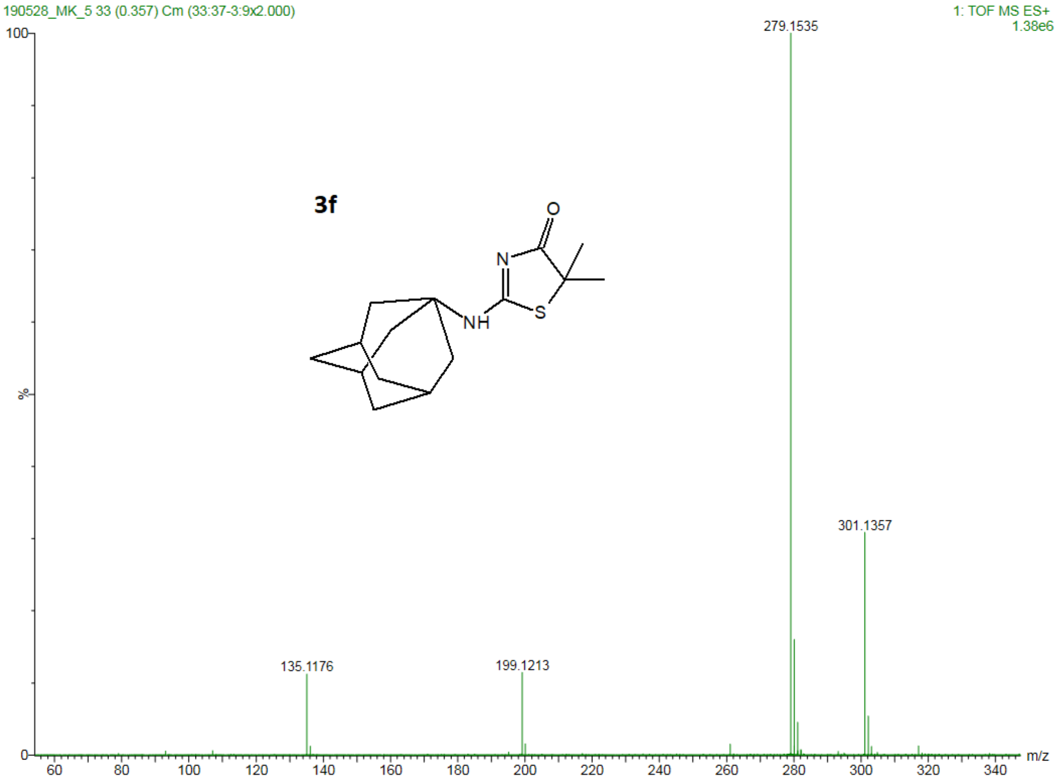

Figure S3F Mass spectrum of compound **3f.**

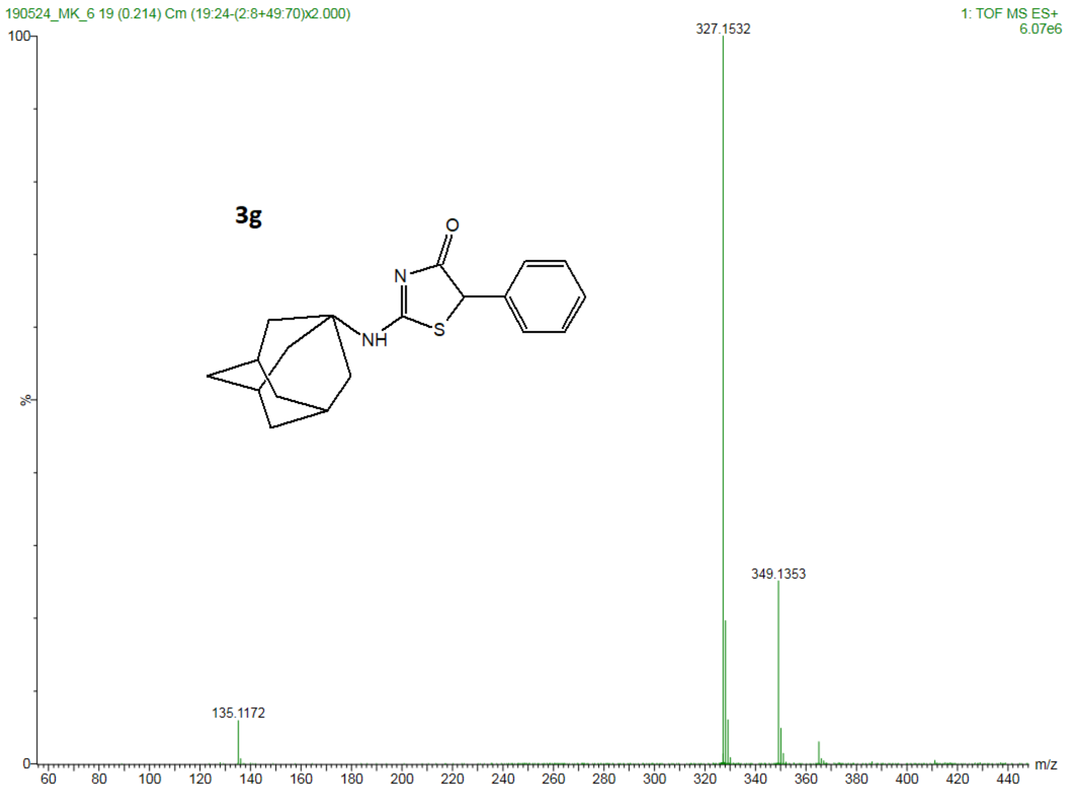

Figure S3G. Mass spectrum of compound **3g.**

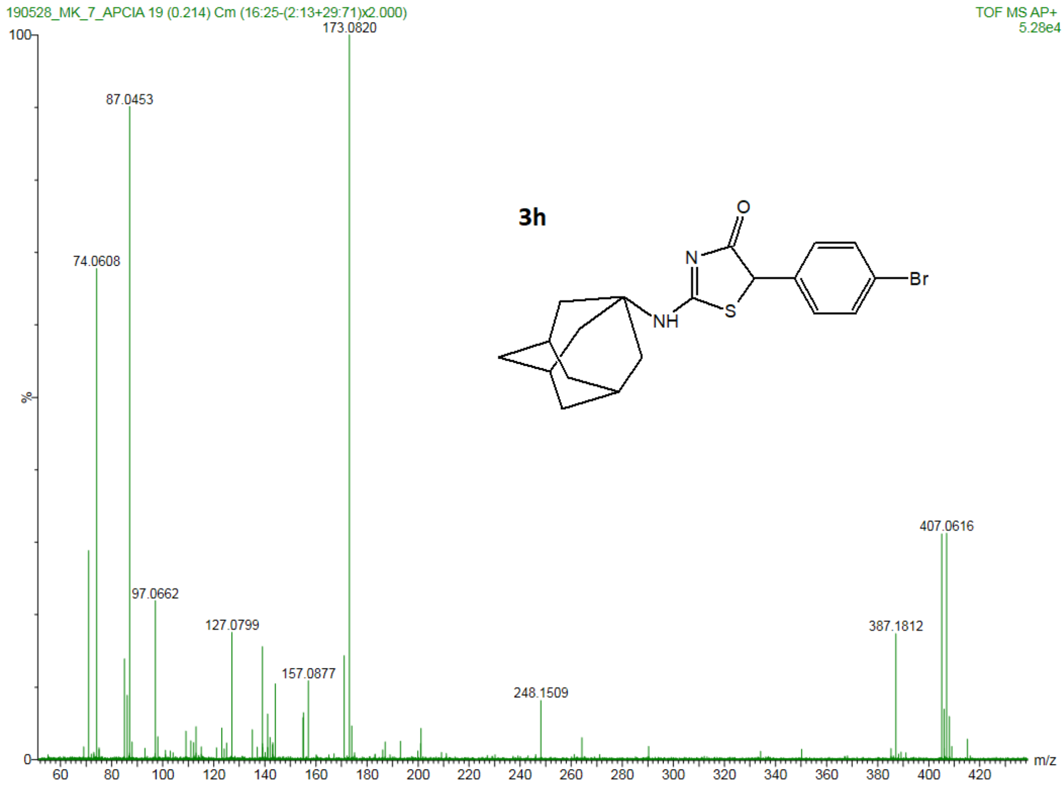

Figure S3H Mass spectrum of compound **3h.**

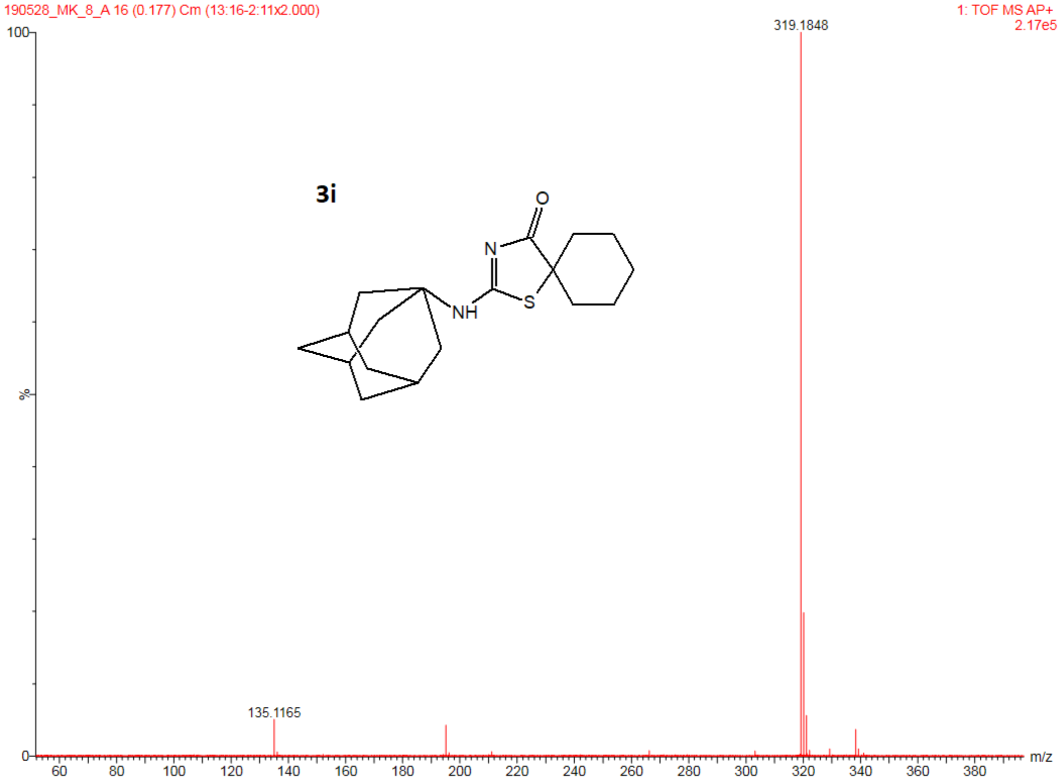

Figure S3I. Mass spectrum of compound **3i.**

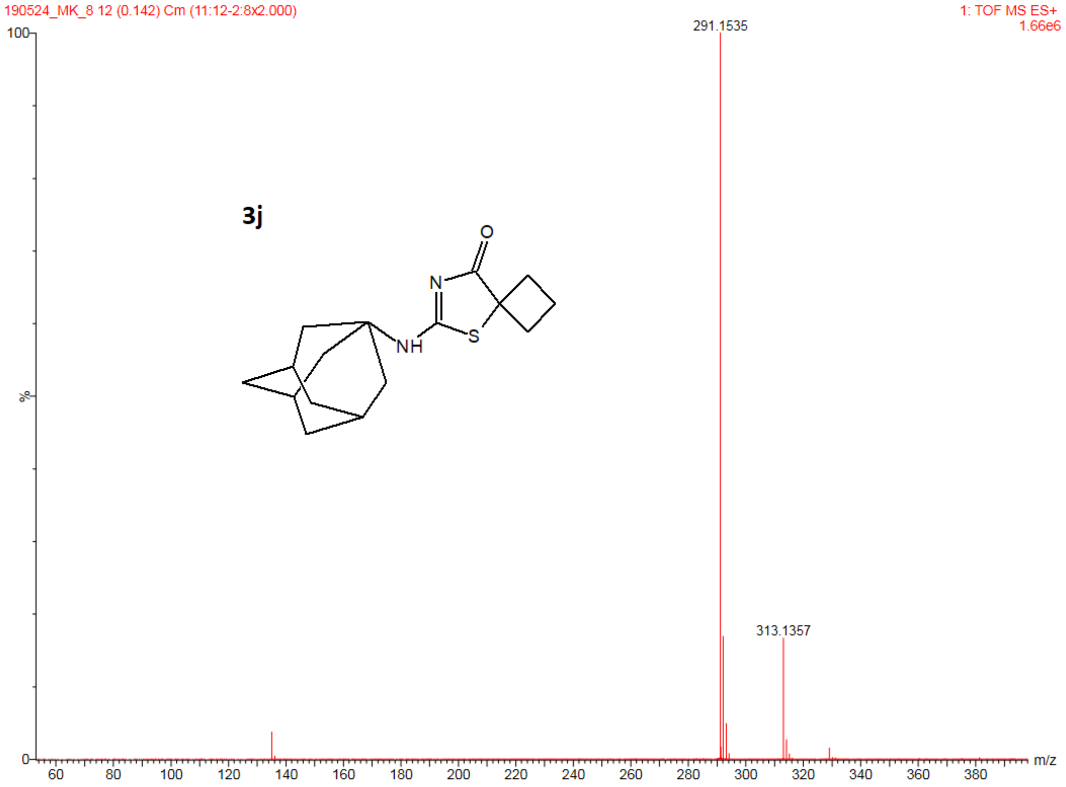

Figure S3J. Mass spectrum of compound **3j.**
